# Machine learning-based identification of CXCL13 and CXCL10 as key biomarkers in rheumatoid arthritis

**DOI:** 10.3389/fbinf.2026.1925964

**Published:** 2026-10-12

**Authors:** Subash M, Sheik Asraf S, Santhanamari M, Nagaraj P, Ayodeji Olalekan Salau, Abdullahi Garba Usman, Mouaz Yousfan

**Affiliations:** 1 Department of Biotechnology, Kalasalingam Academy of Research and Education, Virudhunagar, Tamil Nadu, India; 2 Department of Computer Science and Engineering, School of Computing, SRM Institute of Science and Technology, Tiruchirappalli, Tamil Nadu, India; 3 Department of Electrical/Electronics and Computer Engineering, Afe Babalola University, Ado-Ekiti, Nigeria; 4 Saveetha School of Engineering, Saveetha Institute of Medical and Technical Sciences, Chennai, India; 5 Irfan Suat Gunsel Operational Research Institute, Near East University, Nicosia/TRNC, Türkiye; 6 Department of Pharmacy, Arab International University, Damascus, Syria

**Keywords:** biomarkers, *CXCL10*, *CXCL13*, machine learning, rheumatoid arthritis

## Abstract

**Background:**

Rheumatoid arthritis (RA) is one of the autoimmune diseases that involve joint damage and synovial membrane inflammation. There have been many attempts to examine the utility of biomarkers in the diagnosis of RA, but there is no clinically accepted biomarker for the diagnosis of RA due to its heterogeneity. Thus, in this study, we tried to find RA biomarkers using machine learning and bioinformatics approaches.

**Methods:**

Three corresponding gene expression datasets (GSE55457, GSE12021, and GSE77298) were downloaded from the Gene Expression Omnibus database. Batch effect correction was carried out using the *ComBat* function of the *sva* package in R. Differentially expressed genes (DEGs) compared to controls were identified using *limma*. To identify the biological functions and pathways of DEGs, Gene Ontology, KEGG, and gene set enrichment analysis (GSEA) were conducted. The protein–protein interaction (PPI) network was constructed in Cytoscape using the STRING database. Genes identified through PPI analysis and further filtered by LASSO and SVM-RFE regressions were considered as diagnostic biomarkers. Additional analysis of hub genes was conducted using the GSE89408 dataset. CIBERSORTx was used to evaluate the immune cell infiltration in RA.

**Results:**

A total of 440 DEGs were selected, including 197 downregulated and 243 upregulated genes. The DEGs were involved in biological processes and pathways associated with the immune system and were suggested by functional enrichment analysis. GSEA enriched in cytokine–cytokine receptor interaction, hematopoietic cell lineage, primary immunodeficiency, and RA pathways. The hub genes *CXCL13* and *CXCL10* were chosen with the help of a machine learning algorithm. An external independent validation of the hub genes was conducted. In addition, ROC analysis showed a good diagnostic value of the two genes. The analysis of immune cell infiltration showed *CXCL13* was associated with multiple immune-cell populations, including plasma cells, T-cell subsets, and M1 macrophages, whereas *CXCL10* showed significant associations with M1 macrophages, plasma cells, and selected T-cell subsets. Correlation analysis showed a high correlation between chemokines CXCL13 and CXCL10 and many infiltrated immune cells.

**Conclusion:**

The present study indicates the potential usefulness of *CXCL13* and *CXCL10* genes as diagnostic biomarkers in RA. Synovial inflammation in RA is associated with the coordinated action of many genes involved in the recruitment of various immune cells to the inflammatory site. Although the term chemokines were used to designate them, these genes are the genes encoding molecules associated with the migration of particular immune cell types, and CXCR5 and CXCR3 may be considered the potential targets for future research.

## 1 Introduction

Rheumatoid arthritis is a chronic systemic autoimmune disease characterized by persistent synovial inflammation leading to both local joint damage and systemic manifestations (Firestein, 2003). Synovial hyperplasia in RA is associated with pannus formation, which invades cartilage and bone, ultimately leading to progressive and irreversible joint destruction (Paleolog, 2002). In addition, RA is associated with extra-articular complications affecting organs such as the lungs, heart, and vasculature (Conforti et al., 2021). RA represents one of the leading autoimmune disorders on a global scale, with population-based studies estimating that approximately 0.4%–1% of the global population is affected (Deshmukh, 2023).

The identification of early disease activity and timely intervention is needed to prevent irreversible structural damage in joints and disability. However, currently used biomarkers, including rheumatoid factor and anti-cyclic citrullinated peptide antibodies, demonstrate limited sensitivity and fail to reliably reflect fluctuations in disease activity. These limitations highlight the need for more reliable and robust molecular biomarkers for RA diagnosis and progression monitoring (Schellek et al., 2000; Combe, 2007).

Rapid development in genomic platforms and technologies, such as microarrays, has enabled comprehensive genome-wide expression analysis (Satam et al., 2023). The integration of publicly available repositories such as the Gene Expression Omnibus (GEO) enhances statistical power and reduces batch effects, facilitating robust identification of differentially expressed genes (DEGs) and disease-associated molecular signatures in RA (Edgar et al., 2002).

RA pathogenesis is strongly associated with immune dysregulation within the synovial microenvironment. It is often characterized by T lymphocytes, B lymphocytes, macrophages, and dendritic cells. These resident immune cells secrete a range of pro-inflammatory cytokines, including TNF-α, IL-1β, GM-CSF, and interferon-γ, along with chemokines such as CXCL9, CXCL10, and CXCL13, which are involved in immune cell recruitment and retention in inflamed synovial tissue (Firestein and McInnes, 2017). Here, we used computational approaches such as CIBERSORTx, which have been widely used to characterize immune subset composition from data patterns in RA tissues (Zhou et al., 2021).

In this article, we integrated three gene expression datasets and applied bioinformatics workflows and machine learning classification approaches to identify potential diagnostic biomarkers for RA. Furthermore, we aimed to elucidate the underlying disease mechanisms with a particular focus on immune cell infiltration and characterizing the functional dimension of immune dysregulation operating in the synovial membrane (Yu et al., 2021).

## 2 Materials and methods

### 2.1 Data acquisition

Gene expression datasets (GSE55457, GSE12021, and GSE77298) consisting of synovial tissue samples were retrieved from the GEO repository (https://www.ncbi.nlm.nih.gov/), a widely used open-access platform (Edgar et al., 2002; Clough et al., 2024). Corresponding platform-specific annotation files were also saved for further probe-to-gene mapping. An additional independent dataset (GSE89408), also derived from synovial tissue samples, was used as an independent validation cohort to further evaluate the identified hub genes (Clark et al., 2012). A detailed characteristic of all the GEO datasets is provided in Table 1.

**TABLE 1:** Overview of the GEO datasets used in this study.

Dataset	Platform	Tissue (*Homo sapiens*)	Total samples	Healthy controls (HC)	Rheumatoid arthritis (RA)	Experiment type	Purpose
GSE12021	GPL96	Synovium	21	9	12	Microarray	Training
GSE55457	GPL96	Synovium	23	10	13	Microarray	Training
GSE77298	GPL570	Synovium	23	7	16	Microarray	Training
GSE89408	GPL11154	Synovium	180	28	152	RNA-seq	Independent validation

### 2.2 Data preprocessing and batch-effect correction

By means of the *GEOquery* package in R, the GEO Series Matrix files for three training microarray datasets, GSE12021, GSE55457, and GSE77298, were downloaded. Each dataset was independently processed. The probe IDs were converted to gene symbols using the annotation files of the particular platforms. Unassigned genes that do not have valid gene symbols were removed. For duplicate probes mapping to the same gene, the expression values were averaged using the *avereps()* function of the *limma* package in R (Yang et al., 2022). Only RA and healthy control (HC) samples were chosen for further analysis. The expression datasets of each particular dataset were preprocessed according to the above procedure. After probe annotation, probes without valid gene symbols were removed, and duplicate probes were summarized using the *avereps()* function. No additional low-expression filtering was made, as the GEO Series Matrix files were preprocessed and normalized by the authors of the original studies. A study used the combined dataset from different publications and data sources. Prior to differential expression analysis, the common gene expression matrix was corrected for batch effects using the *ComBat* function from the *sva* R package, with study as a batch variable (Diboun et al., 2006). Principal component analysis (PCA) was performed before and after batch-effect correction to assess the effect of batch correction on the overall expression structure of the samples. The expression matrix after batch-effect correction is used for all further analysis (Chen et al., 2005).

### 2.3 Identification and visualization of differentially expressed genes

DEGs between RA and control synovial tissue samples were identified using the *limma* package (Ritch et al., 2015). A design matrix (RA *versus* control) was built, linear models were fitted to the expression data of all samples *via* the lmFit() function, and eBayes moderation was applied to estimate gene-wise variances (Smyth et al., 2005). DEGs were selected using the criterion of the log2 fold change and adjusted *p*-value according to the Benjamini–Hochberg method. The genes with |log2FC|>1 and adjusted *p*-value < 0.05 were considered as DEGs. Enrichment and network analysis were carried out using DEGs (Li et al., 2025). The volcano plot was created using the *ggplot2* (Wickham, 2016) package, where log2 fold change (log_2_FC) was plotted along the x-axis and negative log_10_ (adjusted *p*-value) was plotted along the y-axis. Genes with |log_2_FC|>1 and adjusted *p*-value < 0.05 were used as the criteria to select DEGs with high differences in gene expression. The heatmap of the top-50 DEGs was created using the *pheatmap* package (Kolde, 2010).

### 2.4 Functional enrichment analysis

Functional enrichment of DEGs was carried out using the *clusterProfiler* package in R (Yu, 2024). Gene symbols of the DEGs were converted into Entrez Gene IDs for subsequent enrichment analysis (Inamo, 2020). We identified enriched DEGs in biological process, molecular function, and cellular component. GO enrichment analysis was performed for the biological process, molecular function, and cellular component terms. KEGG pathway enrichment analysis was also conducted to observe enriched DEGs. The Benjamini–Hochberg method was used for multiple testing correction, with adjusted *p*-value <0.05 considered statistically significant for GO and KEGG enrichment analyses. For GO and KEGG enrichment analyses, gene ratio and the corresponding gene count (Count) were reported for each enriched term or pathway. Gene set enrichment analysis (GSEA) was performed using the *fgsea* algorithm and the *gseKEGG()* function from the *clusterProfiler* package in R, which uses the KEGG database for gene sets. This method was applied to identify the biological characteristics and functions of all the genes in the training set. Genes were ordered based on their logFC scores in a descending order, and 1,000 permutations were run for GSEA (Subramanian et al., 2005; Reimand et al., 2019).

### 2.5 Protein–protein interaction network analysis

To explore potential connections among DEGs, a protein–protein interaction (PPI) network was built. The top-100 DEGs, ranked by absolute logFC, were submitted to the STRING database with a combined interaction score cutoff of ≥0.400 (Szklarczyk et al., 2023), which was viewed and analyzed using Cytoscape. The Cytoscape plugin’s maximum clique centrality (MCC) algorithm was applied to find hub genes (Shannon et al., 2003; Chin et al., 2014).

### 2.6 Machine learning analysis

To refine the candidate hub genes into diagnostic biomarkers, machine learning algorithms were applied (Wu et al., 2024). The training dataset consisted of the combined microarray data that include 41 RA and 26 HC samples. A total of 20 hub genes from the PPI network were selected as the feature set, and the disease status (RA vs. HC) was selected as the response variable.

LASSO (least absolute shrinkage and selection operator) logistic regression was carried out using the *glmnet* package in R (Friedman et al., 2010). A binomial logistic regression model was fitted. To find the optimum regularization parameter (λ), stratified 10-fold cross-validation was performed. Candidate biomarkers were selected based on non-zero regression coefficients; the final model was created using the lambda.1se criterion (λ = 0.1015).

Support vector machine-recursive feature elimination (SVM-RFE) was carried out using the *caret* package along with a linear support vector machine classifier (Kuhn, 2008). Stratified five-fold cross-validation repeated 10 times was performed for all feature sets that contained 1–20 hub genes. The feature set with the highest cross-validation accuracy was chosen as the optimal one. The final hub genes were the genes that overlapped with LASSO and SVM-RFE. All feature selection and modeling were only performed with the training dataset (41 RA and 26 HC). The independent RNA-seq dataset GSE89408 (152 RA and 28 HC) was not involved in feature selection and modeling procedures.

### 2.7 External validation

The potential hub genes obtained in this study were subjected to external validation through an independent RNA-seq dataset, namely, GSE89408. The dataset included 152 RA and 28 HC samples. No class-balancing procedure was applied because this dataset was used only for independent validation. The unequal distribution of RA and HC samples may influence classification-derived performance measures, particularly accuracy, sensitivity, specificity, and F1 score; therefore, the validation results were interpreted primarily based on the ROC-AUC values and were considered supportive rather than definitive evidence of diagnostic performance. Raw read counts were normalized using the *edgeR* package (Chen et al., 2025) by estimating normalization factors and transforming the results into log2 counts per million (CPM). The normalized expression levels of CXCL13 and CXCL10 in RA and HC samples were then analyzed for a consistent expression pattern.

### 2.8 ROC curve analysis

The ROC curve analysis was carried out using the *pROC* package in R (Robin et al., 2011) to determine the diagnostic potential of the detected hub genes. ROC curve was drawn for the *CXCL13* and *CXCL10* genes. The area under the ROC curve (AUC) was used to evaluate the diagnostic potential of the models.

### 2.9 Immune cell infiltration analysis

The relative abundance of 22 immune-cell subsets in RA and healthy control synovium was estimated with CIBERSORTx (https://cibersort.stanford.edu/) (Newman et al., 2019) using the LM22 signature matrix and 1,000 permutations. The batch-corrected microarray expression matrix was used as the input, with quantile normalization enabled and batch correction disabled. The analysis was performed in the relative mode using the log2-transformed batch-corrected microarray expression matrix. All 67 samples satisfied the CIBERSORTx deconvolution quality criterion (*p* < 0.05). Immune cells in the two groups were compared using the Wilcoxon rank sum test, and the resulting *p*-values were adjusted for multiple testing using the Benjamini–Hochberg method. Next, Spearman correlation analysis was performed between the expression levels of CXCL13 and CXCL10 and the estimated fractions of all 22 immune-cell subsets in the RA samples.

## 3 Results

### 3.1 Data integration and batch correction

The three GEO datasets were merged. After annotating probes, eliminating those without valid gene symbols, and summarizing duplicate probes, GSE77298, GSE55457, and GSE12021 retained 22,880, 13,237, and 13,237 unique genes, respectively (Supplementary Table S1), and the flow chart is shown in Supplementary Figure S1. The combined expression matrix included 13,237 common genes for subsequent analyses. To validate the hub genes identified, the RNA-seq dataset GSE89408 served as an independent validation cohort. Figure 1A illustrates that the PCA plot of the combined data revealed a significant batch effect, as the three datasets were separated by study. Post-ComBat normalization, the data demonstrated better sample integration across the studies (B). The distinction between RA and control samples remained evident when the samples were categorized by disease status (RA and healthy control), both prior to (C) and following batch-effect correction (D). The empirical Bayes approach successfully removes batch effect without having much impact on biological variance. Batch-effect correction successfully removed the majority of technical variations, thus leading to greater similarity of samples from different batches while maintaining the biological separation between RA and the control samples. This indicates that *ComBat* effectively minimized technical variability while maintaining the biological signal associated with the disease. Subsequent analyses utilized the expression data that had been normalized and adjusted for batch effects.

**FIGURE 1:**
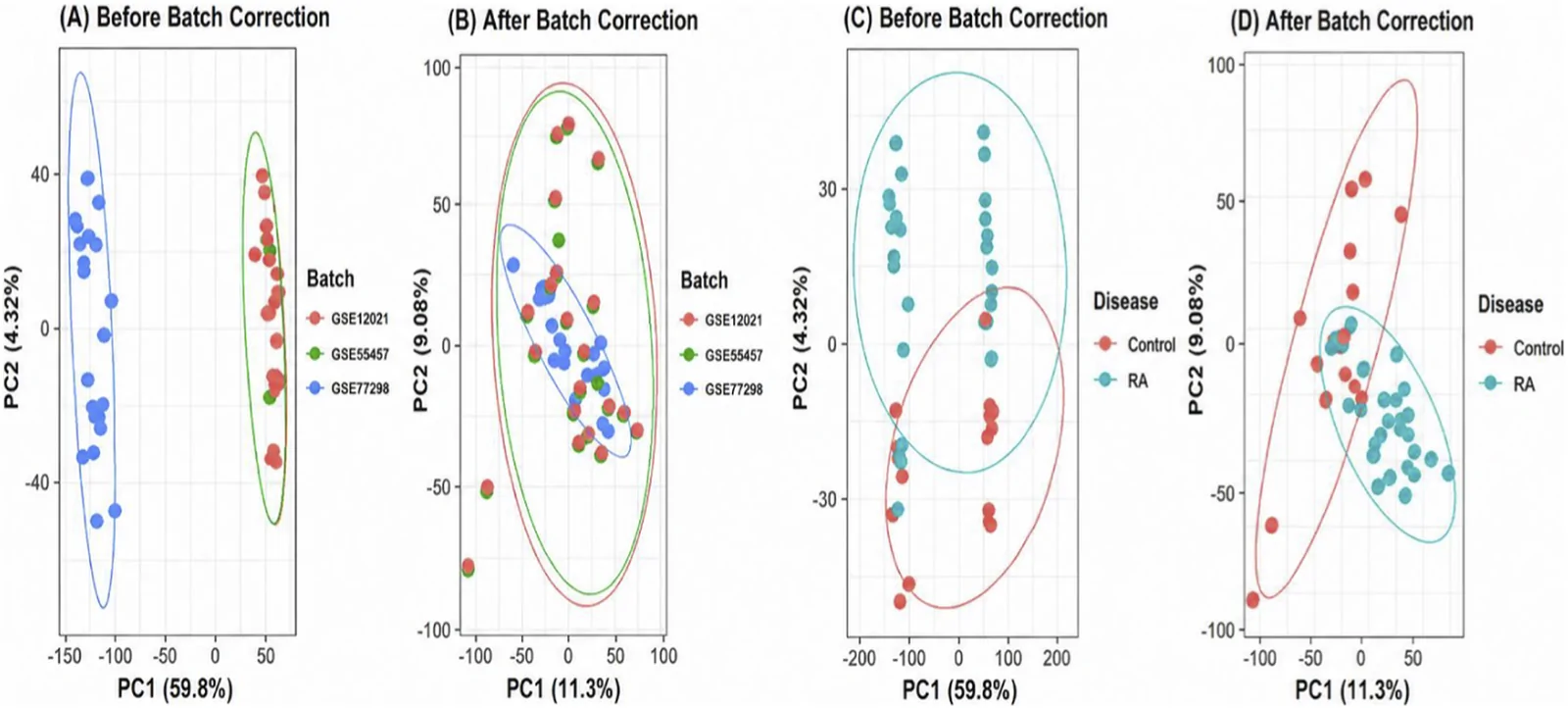
Principal component analysis (PCA) before and after batch-effect correction. PCA results before and after ComBat batch effect correction. **(A)** Before batch-effect correction. **(B)** After ComBat correction. **(C)** Before batch-effect correction in the view of disease status. **(D)** After batch-effect correction in the view of disease status.

### 3.2 Identification of differentially expressed genes

The differential expression analysis conducted using the *limma* package led to 440 DEGs in the comparison of RA and healthy control samples with |log2FC| > 1 and adjusted *p*-value <0.05 after multiple test corrections. Specifically, 243 upregulated genes and 197 downregulated genes were identified in RA synovial tissue. Genes associated with immune response processes were mostly upregulated (for example, chemokines and cytokine receptors), while genes related to metabolism and structural proteins were mostly downregulated. A volcano plot was generated using the *ggplot2* package to visualize the distribution of DEGs, as illustrated in Figure 2 below. The heatmap of the top-50 DEGs was generated using the *pheatmap* package, shown in Figure 3. Gene expression data were scaled, and the genes and samples were hierarchically clustered. As shown in the picture below, the gene expression patterns were clearly distinct between the RA and control samples, with some clustering within each group. Several gene clusters were either upregulated or downregulated in RA samples as compared with that in the control ones, indicating an association of these genes in disease-specific gene expression patterns.

**FIGURE 2:**
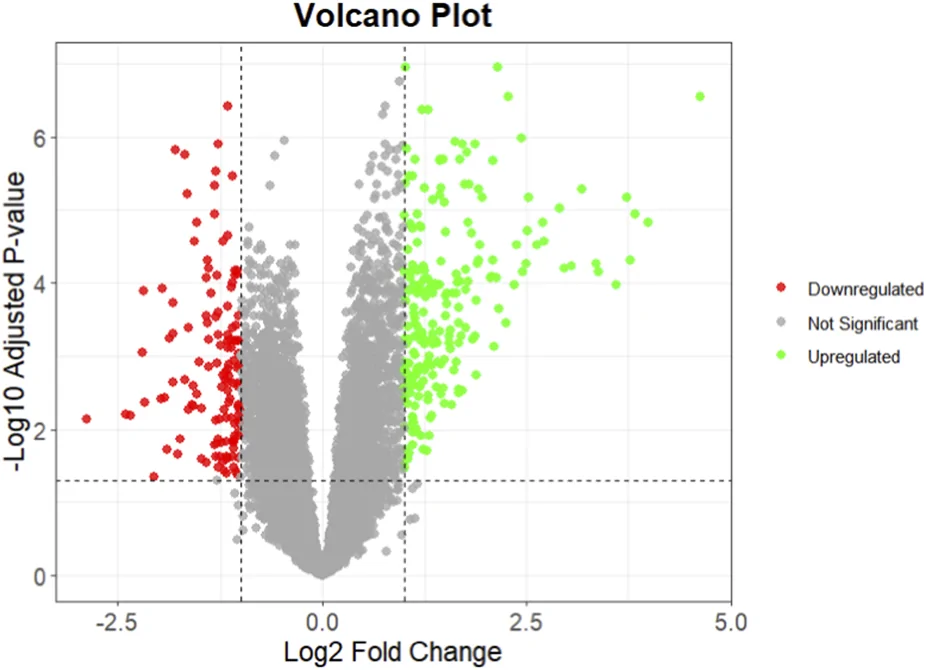
Volcano plot for DEGs between rheumatoid arthritis and healthy controls. Volcano plot for DEGs identified through limma analysis with |log2FC| > 1 and adjusted *p*-value <0.05 as the significance criteria.

**FIGURE 3:**
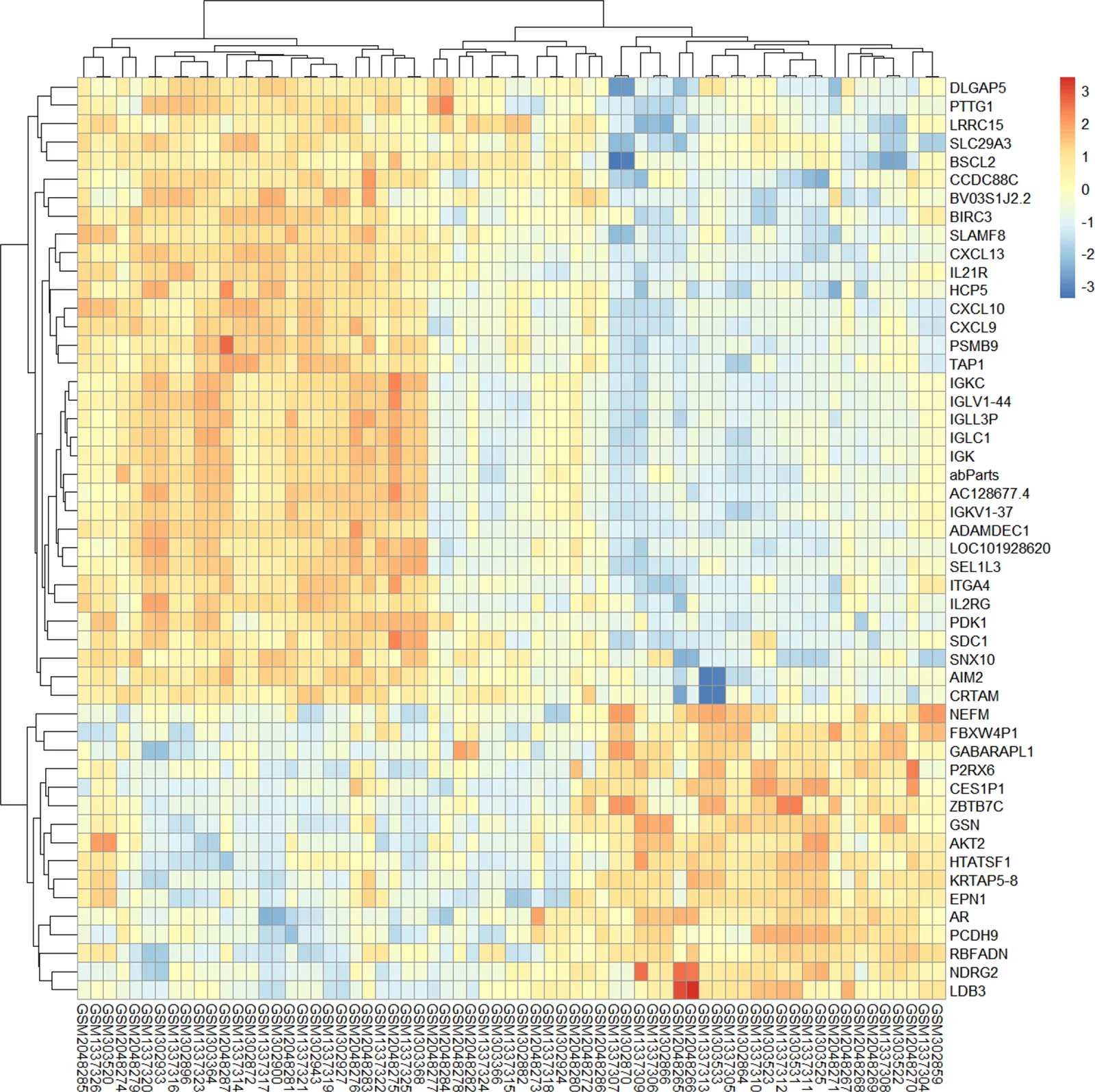
Heatmap of the top-50 DEGs in rheumatoid arthritis and healthy controls. Heatmap of the top-50 DEGs in RA and healthy controls after scaling and hierarchical clustering.

### 3.3 Functional enrichment analysis

The DEGs were significantly enriched in immune-related biological processes, including inflammatory response, T-cell activation, and B-cell receptor signaling pathway. The complete GO enrichment results, including the gene ratio and gene count, are provided in Supplementary Table S2. The proteins predicted to be encoded by DEGs in this category are associated with extracellular signal transduction and immunity. Figure 4. The DEGs were notably enriched in immune-related pathways, including cytokine signaling, cytokine–cytokine receptor interaction, and chemokine-mediated signaling, which are closely associated with RA, as shown by KEGG pathway analysis (Figure 5). The complete KEGG enrichment results, including the p.adj value and gene count, are provided in Supplementary Table S3. GSEA based on the ranked logFC gene list identified several significantly enriched KEGG pathways related to the pathogenesis of RA. The significantly enriched pathways were chosen based on the criteria of *P*-value <0.05 and FDR (q-value) < 0.25, and the top enriched pathways are shown in Figure 6. The complete GSEA results, including gene-set size, normalized enrichment score (NES), p-value, FDR-adjusted p-value, and ranks, are provided in Supplementary Table S4.

**FIGURE 4:**
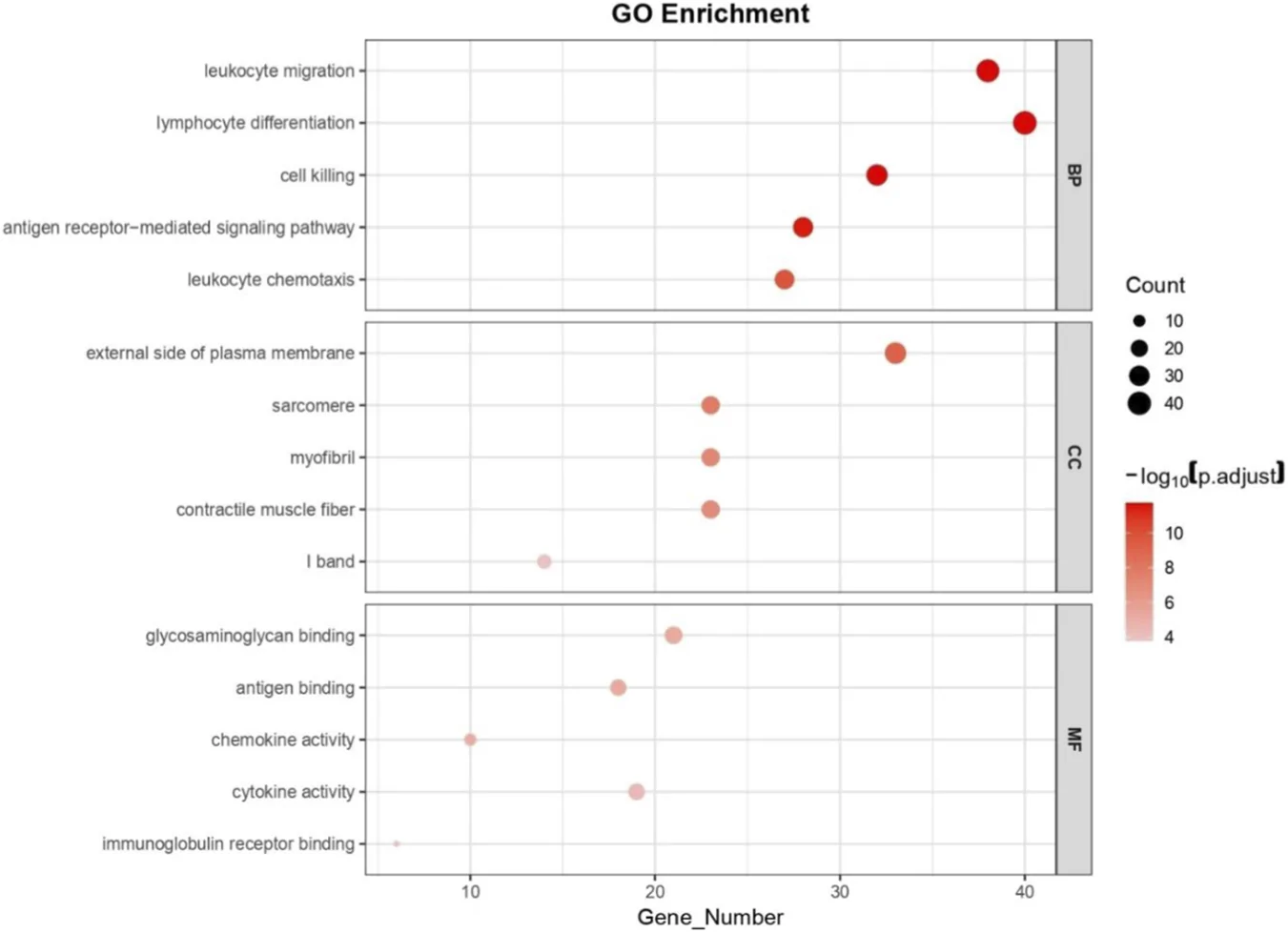
GO enrichment analysis of DEGs. GO enrichment analysis displaying enriched GO categories within DEGs.

**FIGURE 5:**
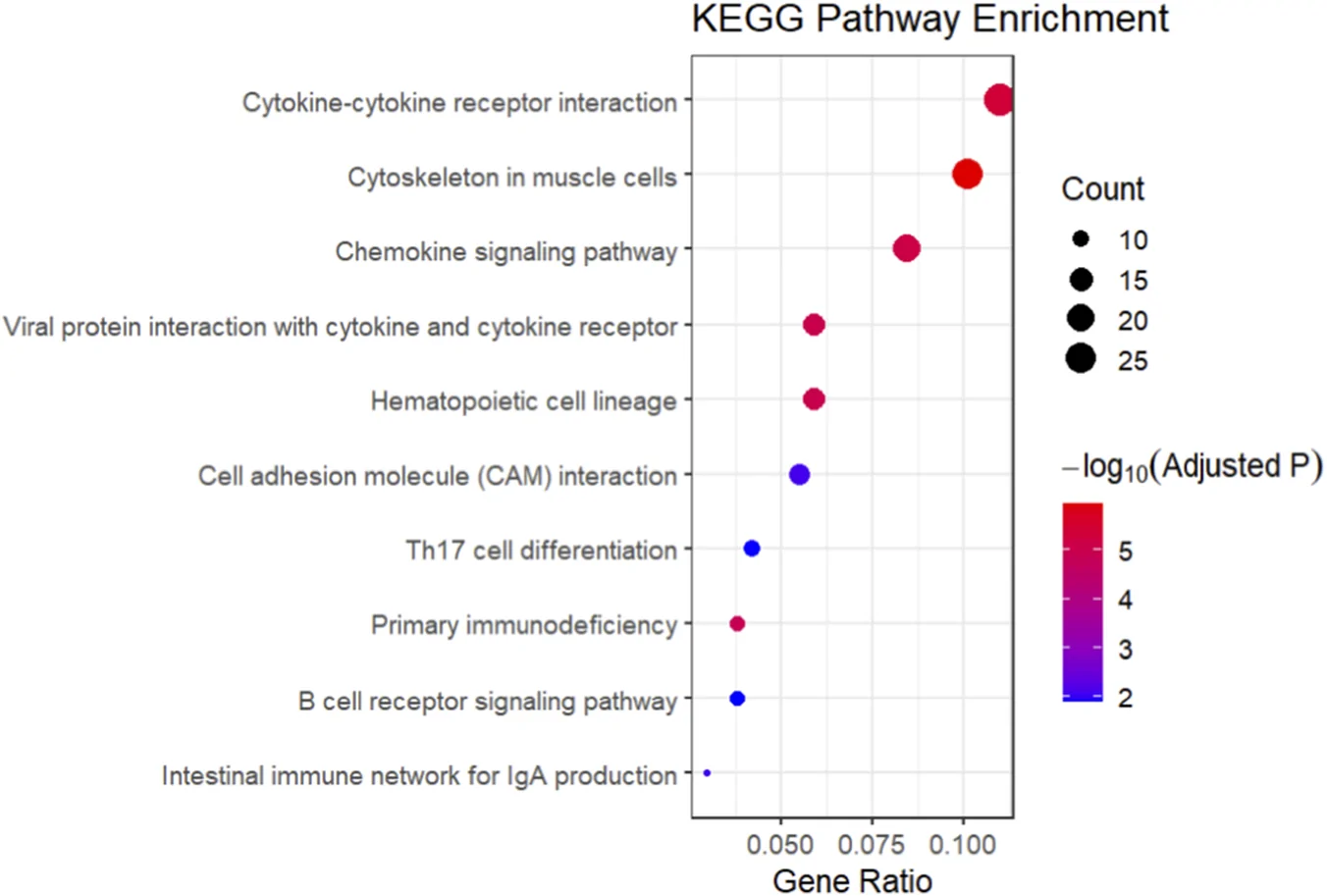
KEGG pathway enrichment analysis of DEGs. KEGG pathway enrichment analysis displaying KEGG pathways related to immune regulation, cytokine signaling, cytokine–cytokine receptor interaction, and chemokine signaling.

**FIGURE 6:**
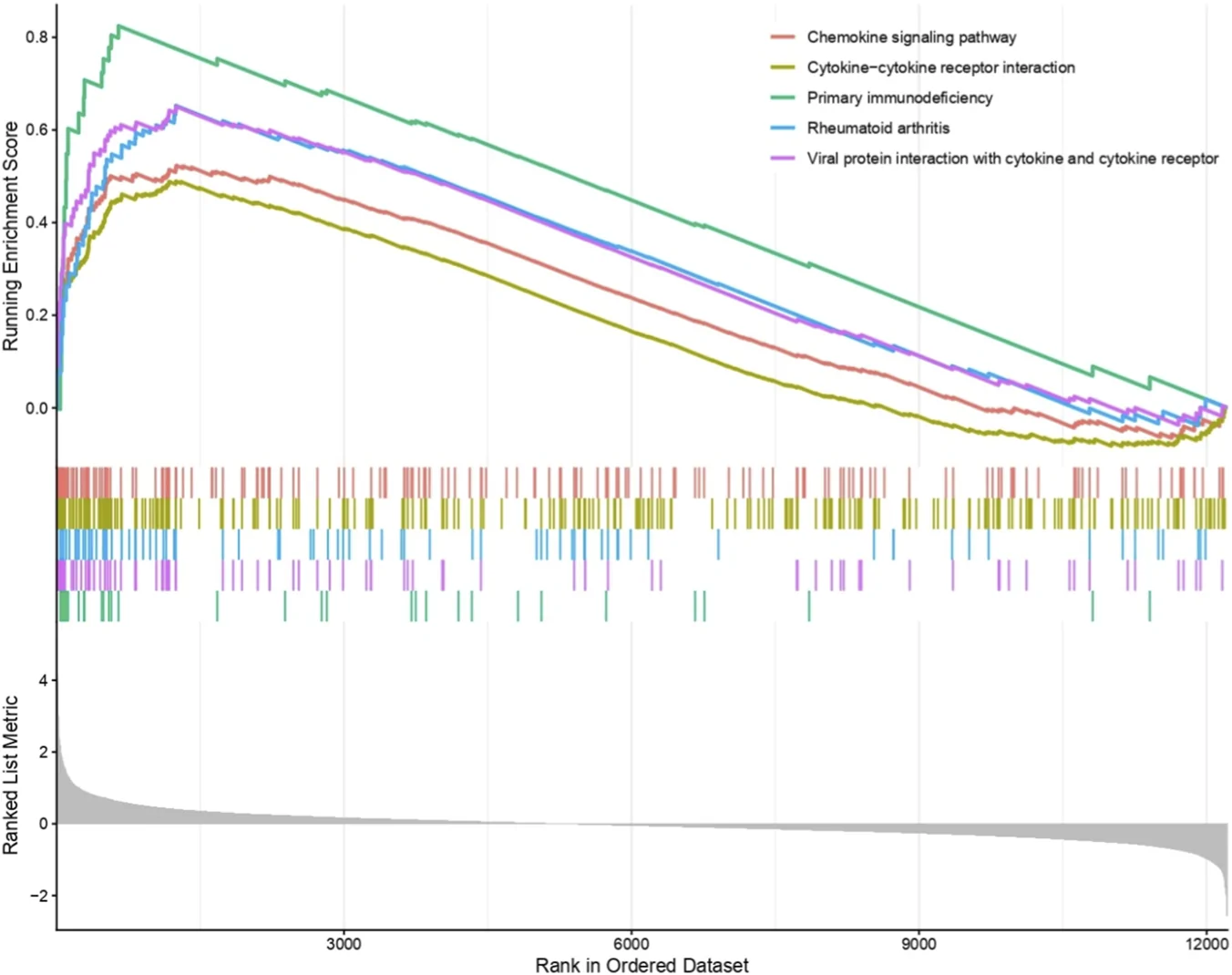
GSEA analysis of the training cohort. GSEA analysis displaying enriched pathways related to immune and inflammation in the training cohort with *p*-value <0.05 and FDR q-value <0.25.

### 3.4 PPI network and hub gene identification

The PPI network was constructed using the top-100 DEGs. The maximum clique centrality (MCC) algorithm implemented in the cytoHubba plugin of Cytoscape was applied to identify hub genes. The top 20 genes ranked by the MCC algorithm were identified as hub genes (Figure 7). The genes are mostly associated with immune cells, chemokine signaling, and inflammatory responses, indicating their crucial roles in RA pathogenesis.

**FIGURE 7:**
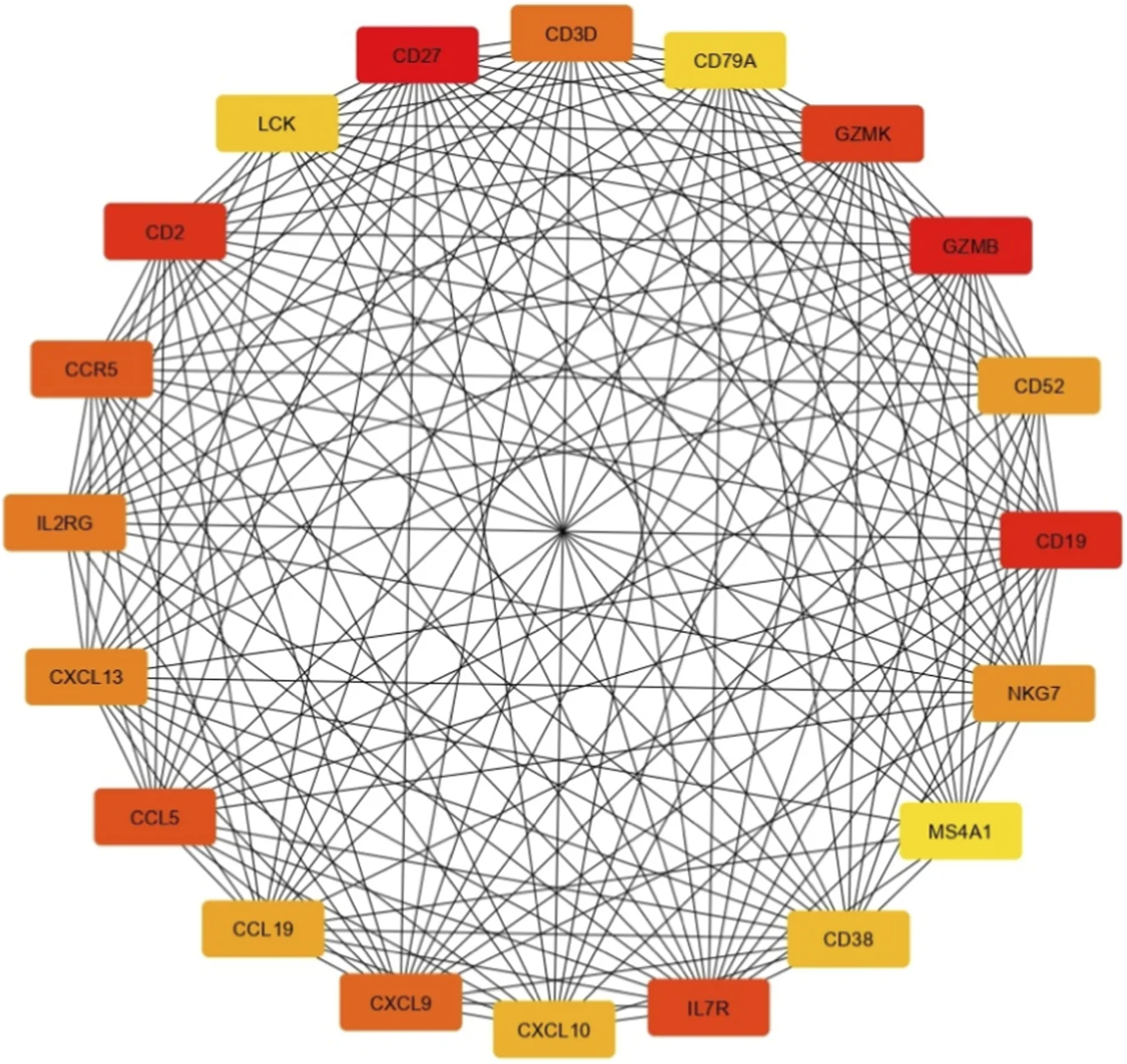
PPI network and hub genes of DEGs. PPI network built with DEGs based on STRING database and hub genes identified using the cytoHubba maximum clique centrality (MCC) algorithm.

### 3.5 Machine learning approaches

Machine learning methods were utilized for the identification of optimal biomarkers from the set of candidate genes derived from the PPI network. LASSO logistic regression analysis was performed for 20 hub genes with the 10-fold cross-validation method. The cross-validation plot depicting the choice of the penalty parameter (λ) is presented in Figure 8A. According to the lambda.1se criterion (λ = 0.1015), only two genes (*CXCL13* and *CXCL10*) having non-zero regression coefficients were kept by LASSO (Supplementary Table S5). Coefficients of the candidate genes at all possible values of λ are depicted in Figure 8B, and it can be seen from the figure that the coefficients of most of the candidate genes shrink to zero with increasing value of λ. Based on the chosen optimal λ from the cross-validation plot (Figure 8A), the final LASSO model was decided, and genes having non-zero coefficients at λ were selected as the optimal LASSO-selected biomarkers.

**FIGURE 8:**
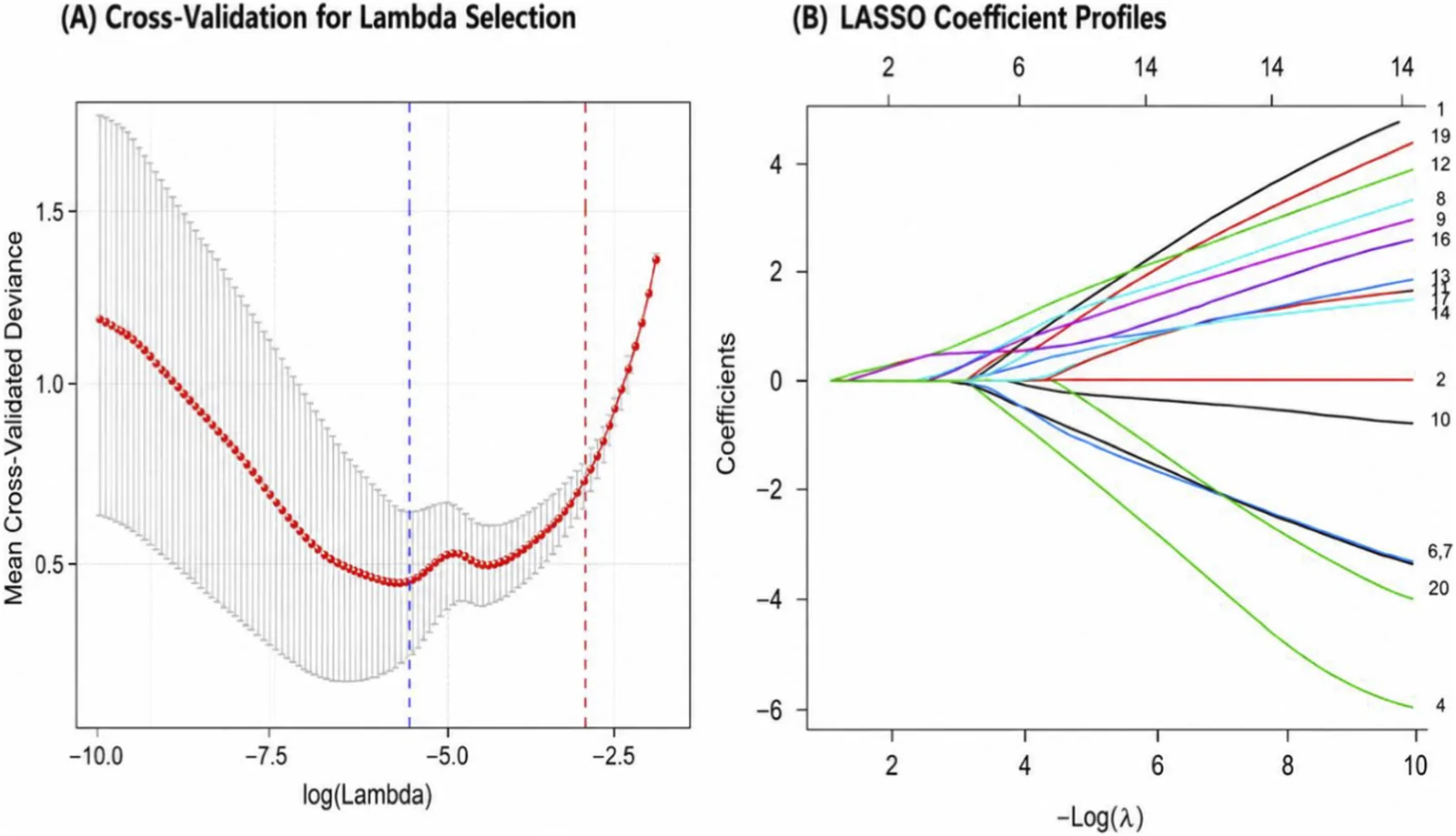
LASSO regression analysis of hub genes. **(A)** Ten-fold cross validation result for selecting optimal λ based on the lambda.1se criterion. **(B)** LASSO coefficient profiles for candidate hub genes, including *CXCL13* and *CXCL10*.

SVM-RFE was performed by a linear SVM classifier with five-fold repeated 10-times cross-validation. Different feature subsets comprising 1–20 hub genes were analyzed, and the optimal set of genes was selected on the basis of maximum cross-validation accuracy (Figure 9). CXCL13 and CXCL10 were selected as the optimal biomarkers according to SVM-RFE analysis (Supplementary Table S6).

**FIGURE 9:**
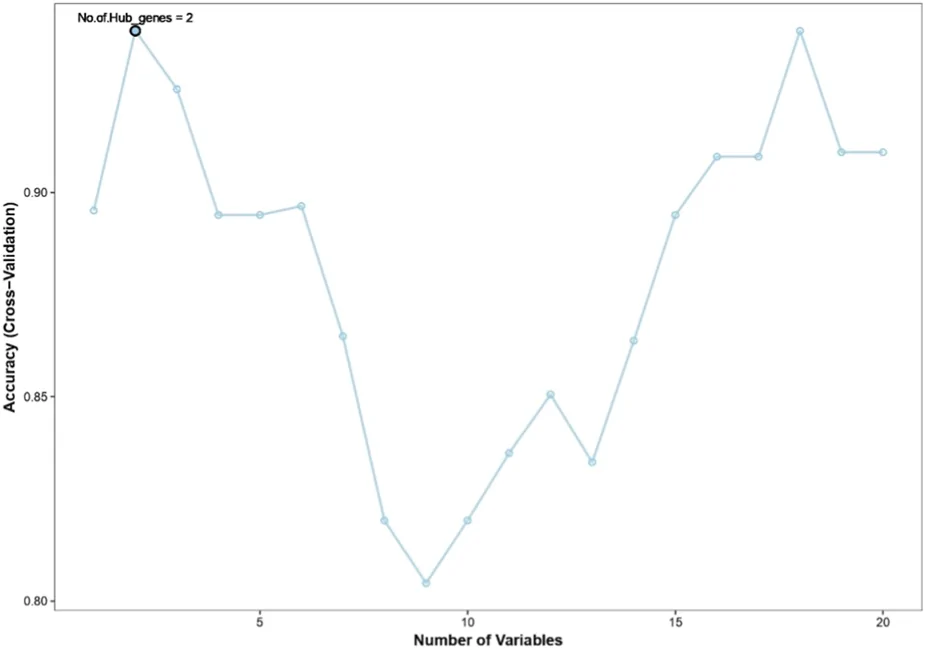
SVM-RFE analysis of candidate hub genes. SVM-RFE analysis for evaluating different features subsets and identification of *CXCL13* and *CXCL10* as the optimal biomarkers.

#### 3.5.1 Identification of final hub genes

The final hub genes were the intersection set of the selected genes from the two machine learning algorithms utilized. *CXCL13* and *CXCL10* were identified by both the LASSO and SVM-RFE algorithms; they are the potential candidate diagnostic biomarkers of the present study. These biomarkers were then evaluated in an independent RNA-seq dataset GSE89408.

### 3.6 ROC analysis of candidate biomarkers

After ROC curve analysis of the identified biomarkers, CXCL13 produced an AUC value of 0.954, an accuracy value of 89.6%, a sensitivity of 85.4%, a specificity of 96.2%, and an F1 score of 90.9% in the training set. CXCL10 produced an AUC value of 0.925, an accuracy value of 88.1%, a sensitivity of 87.8%, a specificity of 88.5%, and an F1 score of 90.0% in the training set. The ROC curve of the biomarkers in the training set is presented in Figure 10, while the expression profile is presented in Figure 11.

**FIGURE 10:**
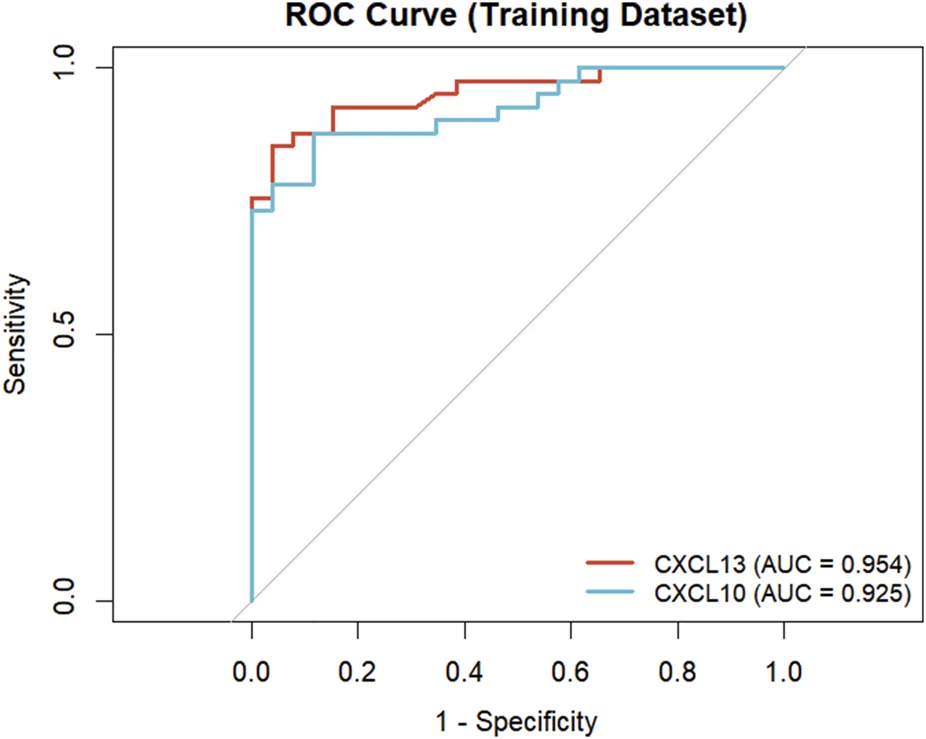
ROC analysis of the identified hub genes in the training dataset. ROC curves showing the diagnostic performance of *CXCL13* and *CXCL10* for distinguishing RA from healthy controls in the training dataset.

**FIGURE 11:**
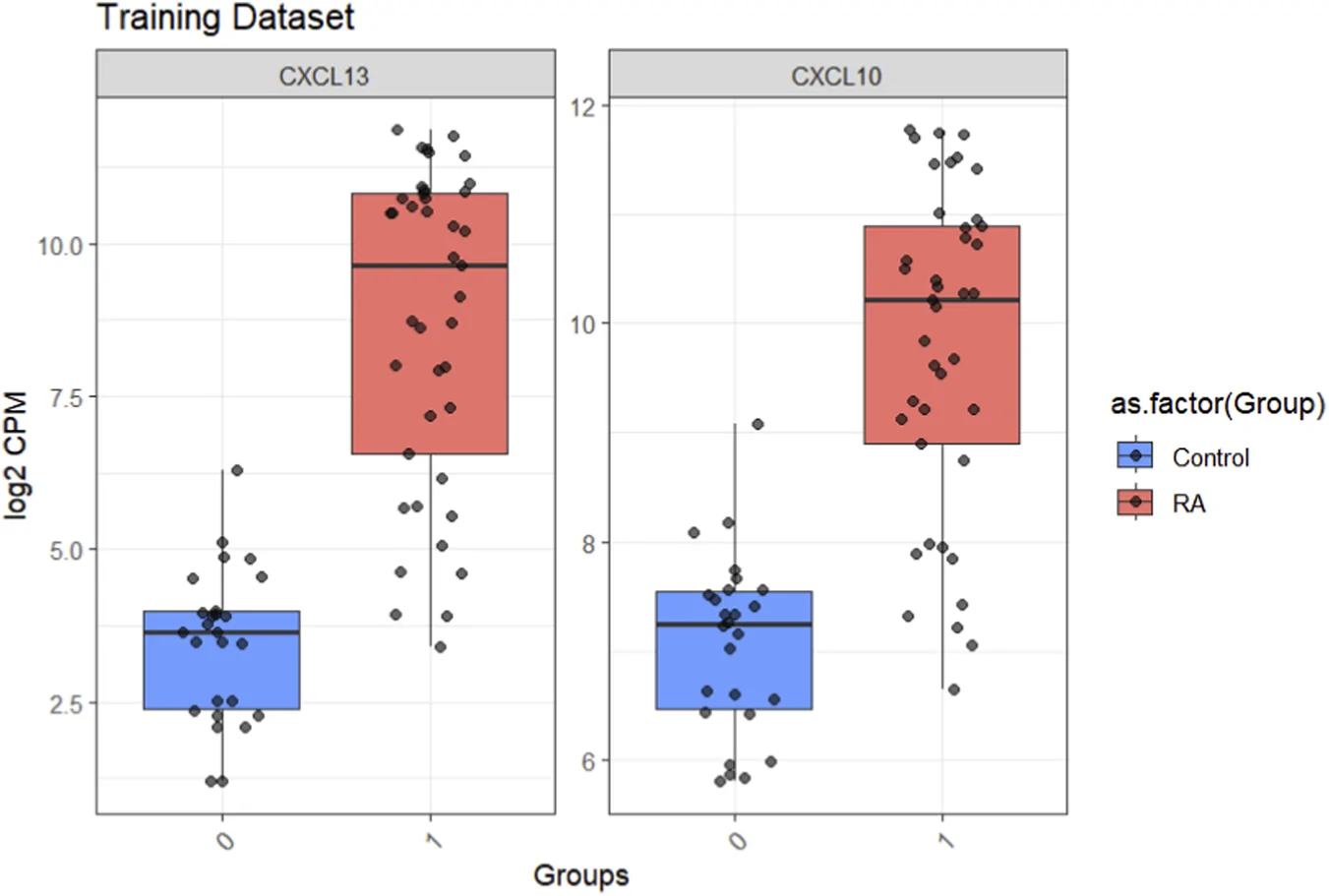
Expression levels of *CXCL13* and *CXCL10* in the training dataset. Expression profiles of the identified hub genes between RA and healthy control samples.

After evaluating the biomarkers using the independent RNA-seq validation dataset (GSE89408), CXCL13 produced an AUC of 0.901, an accuracy of 81.7%, a sensitivity of 80.9%, a specificity of 85.7%, and an F1 score of 88.2%. CXCL10 produced an AUC of 0.886, an accuracy of 78.9%, a sensitivity of 75.7%, a specificity of 96.4%, and an F1 score of 85.8%. The ROC curve of the biomarkers in the validation set is presented in Figure 12, while the expression profile is presented in Figure 13.

**FIGURE 12:**
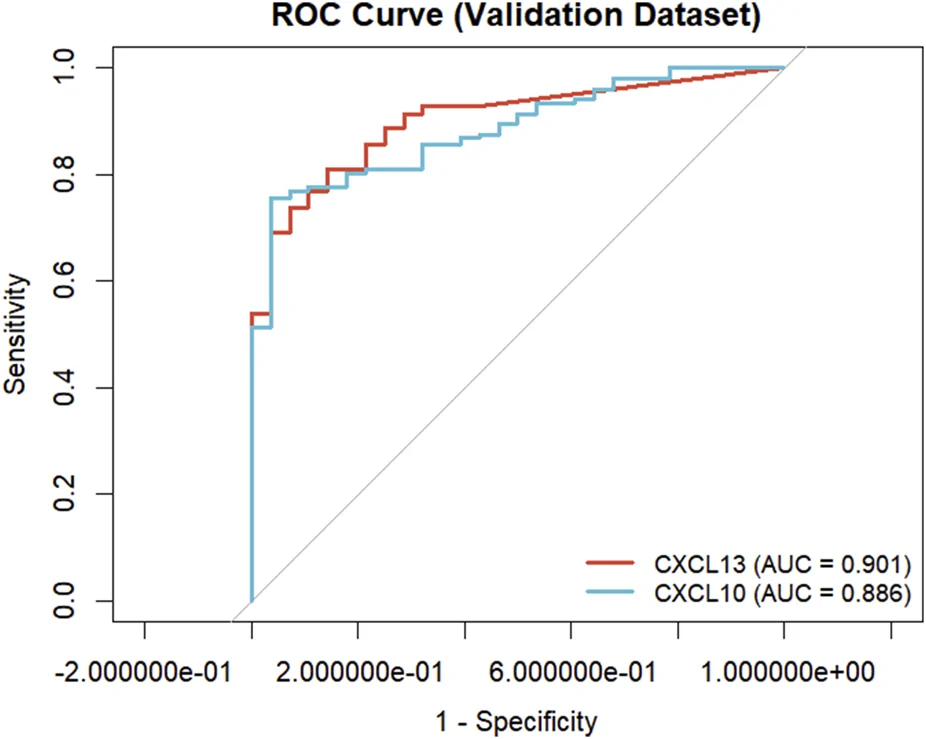
ROC analysis of the identified hub genes in the external validation dataset. ROC curves showing the diagnostic performance of *CXCL13* and *CXCL10* for distinguishing RA from healthy controls in the independent RNA-seq validation dataset GSE89408.

**FIGURE 13:**
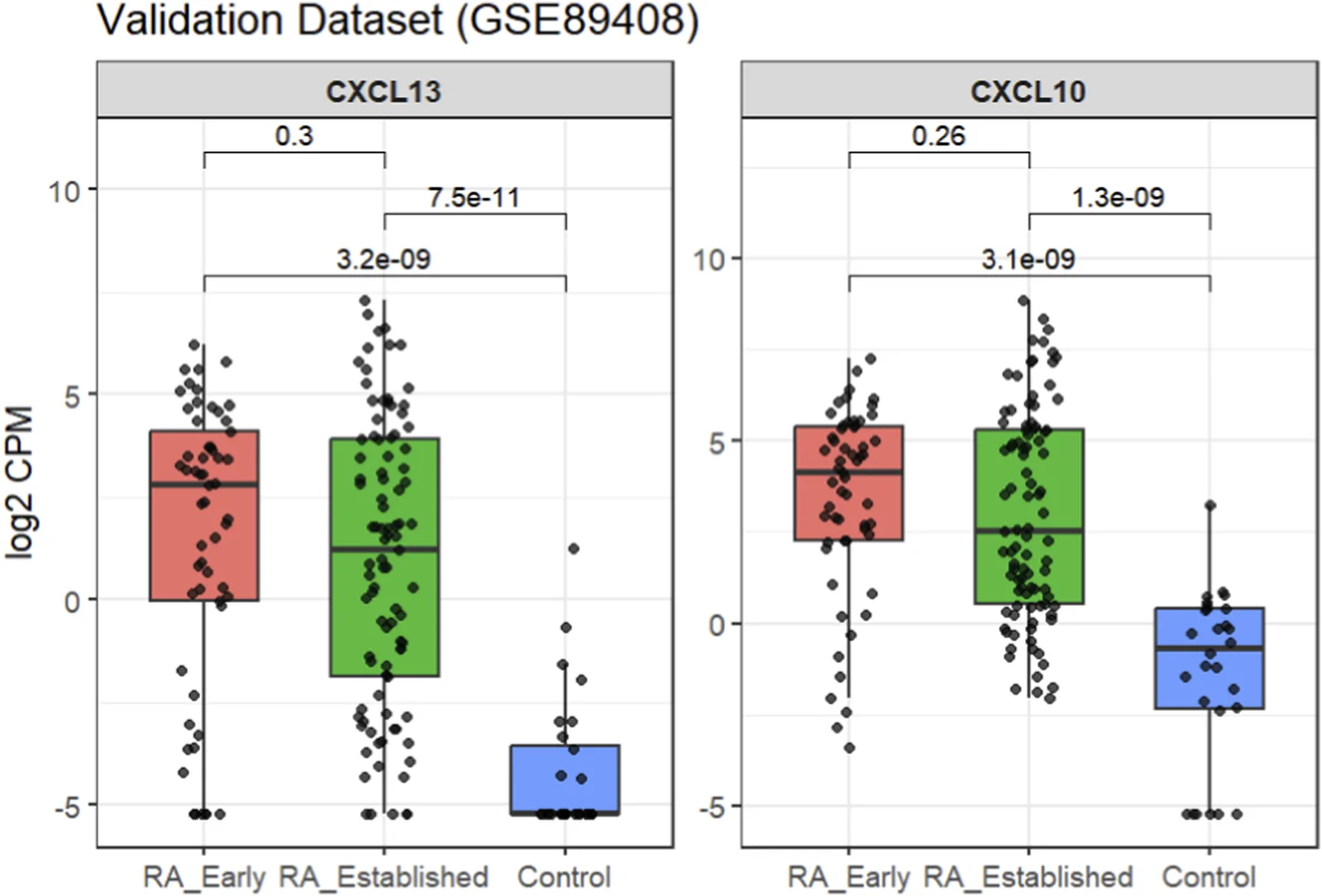
Expression levels of *CXCL13* and *CXCL10* in the external validation dataset. Expression profiles of the identified hub genes among RA and healthy control samples in GSE89408.

The AUC values of the biomarkers in both the training and validation datasets are summarized in Table 2. Although the performance of the biomarkers was lower in the validation set than in the training set, they still retained good diagnostic performance. This shows the potential use of CXCL13 and CXCL10 as diagnostic biomarkers for RA. A detailed summary of the performance metrics, including accuracy, sensitivity, specificity, precision, and F1 score for both datasets, is provided in Supplementary Table S7.

**TABLE 2:** Diagnostic performance of the identified hub genes in the training and validation datasets. AUC values of *CXCL13* and *CXCL10* in the training dataset and the independent validation dataset GSE89408.

Model type	Training AUC value	Validation AUC value
*CXCL13*	0.954	0.901
*CXCL10*	0.925	0.886

### 3.7 Immune cell infiltration in RA synovium

Figure 14 depicts the immune cell infiltration profile analyzed by CIBERSORTx based on the LM22 signature in the samples of RA patients and healthy individuals. Immune cell infiltration in the synovium of RA patients and healthy individuals differed. Compared to healthy subjects, the synovium of RA patients showed significant differences in plasma cells, T cells follicular helper, gamma delta T cells, monocytes, M1 macrophages, as well as decreased infiltration of resting mast cells. The mean immune cell fractions for the RA and control groups are presented in Supplementary Table S10, while the significantly altered immune cell populations are summarized in Supplementary Table S9. Importantly, the hub genes identified exhibited significant associations with different immune cell infiltrates analyzed by CIBERSORTx. CXCL13 was associated with multiple immune-cell populations, including plasma cells, T-cell subsets, and M1 macrophages, whereas CXCL10 showed significant associations with M1 macrophages, plasma cells, and selected T-cell subsets. These differences in immune cell infiltration between the two groups are illustrated in Figure 15.

**FIGURE 14:**
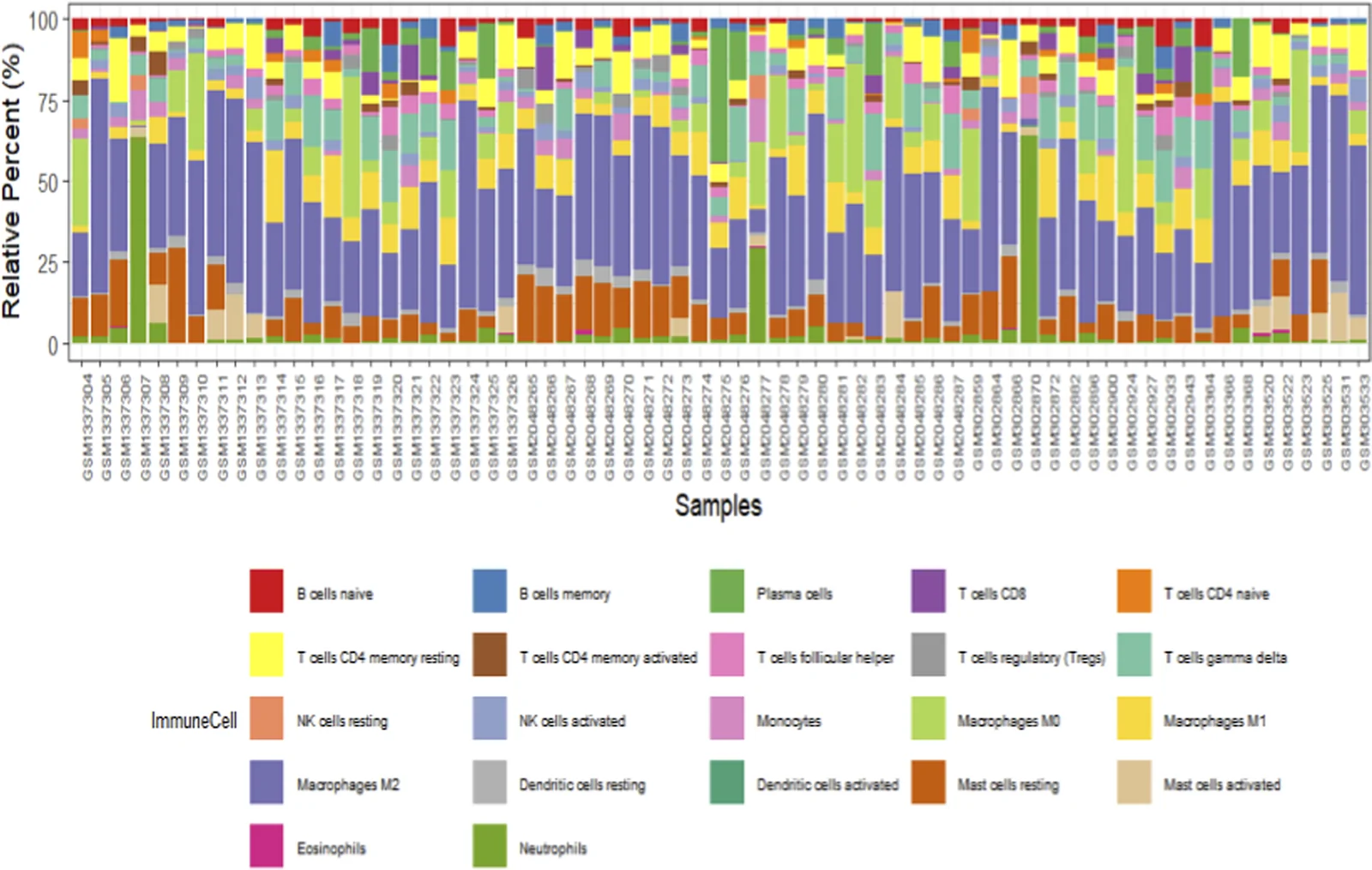
Relative proportions of 22 immune cells in rheumatoid arthritis and healthy controls estimated using CIBERSORTx. Relative proportions of 22 immune cell subsets in RA and healthy control synovial tissue samples estimated using LM22 signature matrix.

**FIGURE 15:**
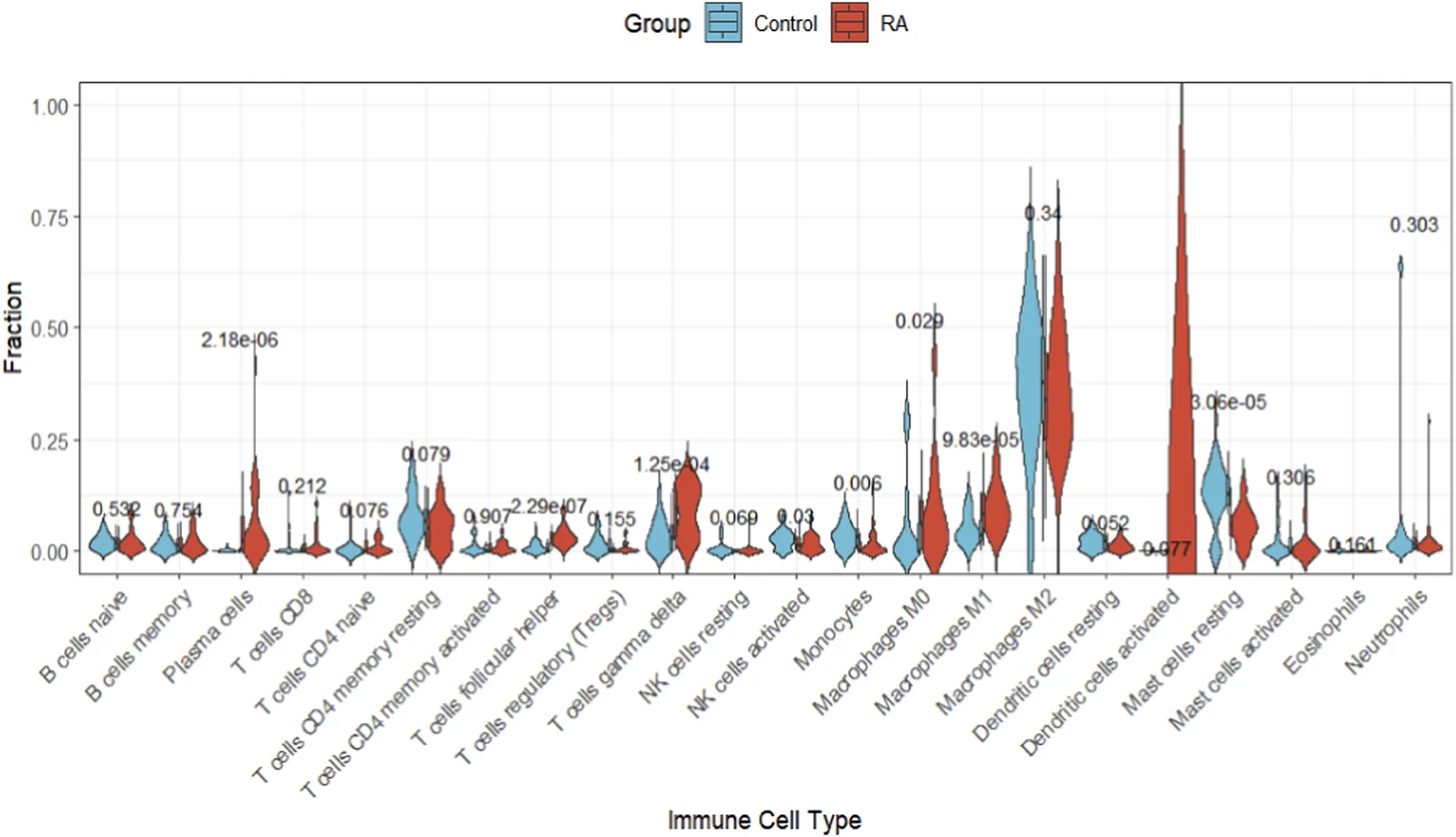
Comparison of immune cell infiltration between rheumatoid arthritis and healthy control groups. Violin plots of difference in the estimated proportions of 22 immune cell subsets between RA and healthy controls.

### 3.8 Hub genes and immune cells correlation analysis

Correlation analysis between RA-associated hub gene expression and immune cell infiltration was conducted on the RA cohort using estimates of the fractions of 22 immune cell populations and the expression of CXCL10 and CXCL13 (Figure 16). Multiple testing correction using the Benjamini–Hochberg procedure identified significant positive correlations between CXCL13 expression and M1 macrophages (r = 0.75) and between CXCL10 expression and M1 macrophages (r = 0.83), and these along with complete correlation results across all 22 immune cell populations are provided in Supplementary Table S11. The strongest significant correlations are presented in Supplementary Table S8 and summarized in Table 3.

**FIGURE 16:**
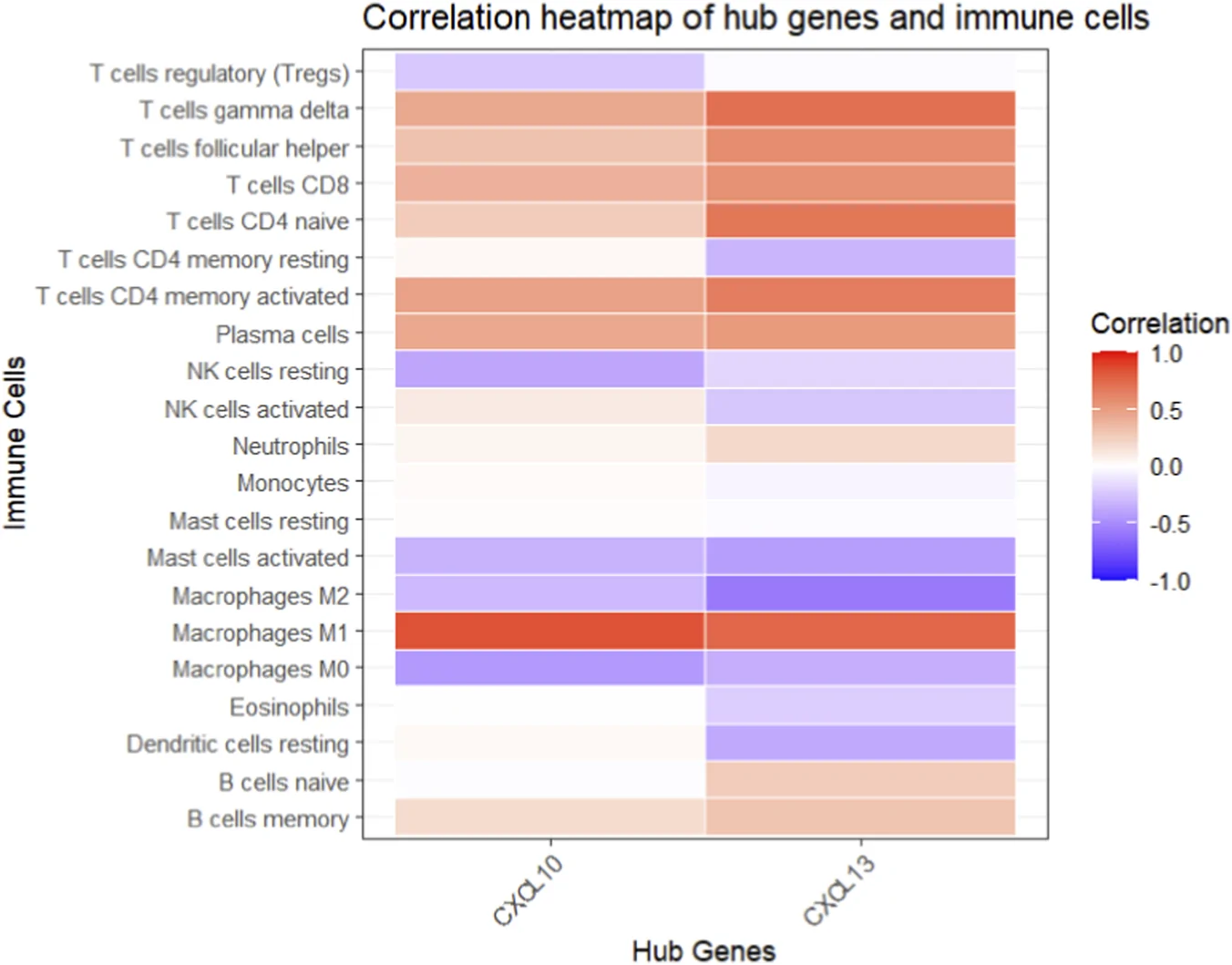
Correlation between hub gene expression and immune cell infiltration in rheumatoid arthritis. Spearman correlation heatmap of CXCL13 and CXCL10 expression levels and estimated proportions of immune cell subsets in the RA cohort.

**TABLE 3:** Top correlations between hub gene expression and immune cell infiltration in rheumatoid arthritis samples.

Gene	Cell	Correlation
*CXCL10*	Macrophages M1	0.833101
*CXCL13*	Macrophages M1	0.751916
*CXCL13*	T cells gamma delta	0.717596
*CXCL13*	T cells CD4 naive	0.680424

## 4 Discussion

RA is a long-term inflammatory condition that targets the immune system and produces discomfort in the joints in the hands and legs. In the initial stage, the joint lining (synovial membrane) is affected. In addition, despite advances in understanding the molecular mechanisms underlying the disease, identification of reliable early diagnostic biomarkers remains limited, mainly due to disease heterogeneity (Paleolog, 2002). Moreover, we used integrative bioinformatics and machine learning approaches to generate an analytical platform that can be used to identify and validate potential biomarkers for the diagnosis of RA and elucidate their associated biological pathways (Yang et al., 2022).

After combining the gene expression data, 440 genes were significantly identified. The application of batch-effect correction before differential expression analysis improved the integration of the three independent datasets while preserving the biological differences between RA and HC samples, thereby increasing the reliability of the downstream analyses. By combining gene expression data from RA and control samples, the results revealed greater stability and reduced variance, including across experimental settings. Additionally, DEGs in RA and control samples are clearly distinguishable, including gene expression changes in RA. By using functional enrichment analysis methods (GO, KEGG, and GSEA) (Reimand et al., 2019), we also identified that DEGs were enriched in the chemokine signaling pathway, cytokine–cytokine receptor interaction, primary immunodeficiency, and hematopoietic cell lineage. By constructing and analyzing the PPI network (Shannon et al., 2003), we identified 20 genes that were significantly involved in gene interactions. Chemokine activity and immune cell activation were the main functions of these genes. Two different machine learning algorithms (LASSO and SVM-RFE) (Friedman et al., 2010; Kuhn, 2008) were applied to improve the robustness of feature selection and minimize the risk of overfitting. The use of two complementary feature-selection algorithms reduced the likelihood of selecting dataset-specific biomarkers and increased confidence in the final candidate genes identified by both methods. As a result, the hub genes *CXCL13* and *CXCL10* remained in the network. An independent test dataset was used to validate the expression of these genes in RA. ROC curve analysis of these genes indicated that they are promising biomarkers for RA diagnosis (Robin et al., 2011).

Furthermore, we deployed CIBERSORTx (Newman et al., 2019) to determine immune cell infiltration in RA patients’ synovial tissues. Compared with the controls, RA synovial tissues show increased numbers of plasma cells, M1 macrophages, and γδ T cells but decreased numbers of resting mast cells, monocytes, and NK cells. The correlation between the hub genes and immune cells was verified by correlation analysis (Lu et al., 2025). *CXCL13* and *CXCL10* had a significant association with activated immune cells, such as macrophages and plasma cells, but inversely correlated with resting immune cells. These results indicate that *CXCL13* and *CXCL10* are associated with immune cell infiltration patterns. *CXCL13* has been reported to be associated with B-cell migration and is also involved in the formation of ectopic lymphoid structures in the synovial tissue. *CXCL10* has been reported to be associated with T-cell migration and macrophages during inflammation. Meanwhile, these genes were also closely related to the movement of leukocytes, cytokine signal transduction, and interactions between leukocytes, which were all closely related to the pathogenesis of RA (Choy, 2012; Zlotnik and Yoshie, 2000).

The CXCL13–CXCR5 and CXCL10–CXCR3 signaling axes may represent potential target pathways that warrant further investigation. The CXCL13–CXCR5 pathway is important for B-cell migration (Pan et al., 2022). The CXCL10–CXCR3 pathway is important for the migration of T cells and macrophages (Yue et al., 2024). Inhibiting CXCR3 and CXCR5 receptors on immune cells may also block activation by other ligands within the multi-ligand, multi-receptor families of chemokines. This study is based on the analysis of gene expression using microarray analysis, which may have inherent limitations. We found that the levels of the two chemokines CXCL13 and CXCL10 were high in patients with RA and associated with immune dysfunction. As these two proteins are highly relevant and valuable for the diagnosis of RA, their corresponding receptor axes (CXCR5 and CXCR3) may represent potential hypotheses for future investigation (Zhao et al., 2021). It is worth noting that CXCL13 and CXCL10 have previously been examined in RA, including as baseline serum predictors of response to TNF-inhibitor therapy in a small prospective clinical cohort (Han et al., 2016). The present study differs in both the aim and approach: rather than a treatment-response biomarker measured in peripheral blood, we establish CXCL13 and CXCL10 as diagnostic biomarkers derived from synovial tissue transcriptomics, using an integrative framework that combines batch-corrected multi-cohort gene expression data, dual machine-learning feature selection (LASSO and SVM-RFE), and external validation in an independent RNA-seq dataset. We further link these biomarkers to specific immune cell infiltration patterns *via* CIBERSORTx deconvolution, providing a mechanistic dimension that prior single-cohort protein studies did not address. Overall, this work highlighted CXCL13 and CXCL10 as possible biomarkers for diagnostic and potential future exploration of RA.

## 5 Conclusion

This research explored the possible novel biomarkers and pathways for RA using bioinformatics and machine learning methodologies. DEGs were first identified by integrating various gene expression patterns and reducing batch effects (Wu et al., 2024). DEGs were implicated in immune-related biological processes, including the interferon signaling pathway, inflammatory responses, and cytokine–cytokine interaction, according to functional enrichment analysis, indicating a strong relationship between immunological dysfunction and the development of RA. According to protein–protein interaction network analysis and machine learning (LASSO and SVM-RFE) techniques (Wu et al., 2024), *CXCL13* and *CXCL10* were hub genes that were upregulated in RA synovial tissues and showed a high area under the ROC curve (AUC).

We identified the biological processes linked to the gene expression profile using immune infiltration analysis, including a pro-inflammatory state due to high concentrations of activated immune cells, including macrophages and plasma cells. The potential involvement of chemokines such as CXCL13 and CXCL10 in RA was assessed using correlation analysis (Han et al., 2016). However, the receptor pathways through CXCR5 and CXCR3 may be studied as potential targets for future research (Zhao et al., 2021).

## Data Availability

The datasets analyzed in this study are publicly available in the NCBI Gene Expression Omnibus (https://www.ncbi.nlm.nih.gov/geo/) under accession numbers GSE55457, GSE12021, GSE77298 and GSE89408.

## References

[B1] Chen L. Borozan I. Feld J. Sun J. Tannis L.-L. Coltescu C. (2005). Hepatic gene expression discriminates responders and nonresponders in treatment of chronic hepatitis C viral infection. Gastroenterology 128, 1437–1444. 10.1053/j.gastro.2005.01.059 15887125

[B2] Chen Y. Chen L. Lun A. T. L. Baldoni P. L. Smyth G. K. (2025). edgeR v4: powerful differential analysis of sequencing data with expanded functionality and improved support for small counts and larger datasets. Nucleic Acids Res. 53, 53. 10.1093/nar/gkaf018 39844453 PMC11754124

[B3] Chin C.-H. Chen S.-H. Wu H.-H. Ho C.-W. Ko M.-T. Lin C.-Y. (2014). cytoHubba: identifying hub objects and sub-networks from complex interactome. BMC Syst. Biol. 8, S11. 10.1186/1752-0509-8-S4-S11 25521941 PMC4290687

[B4] Choy E. (2012). Understanding the dynamics: pathways involved in the pathogenesis of rheumatoid arthritis. Rheumatology 51, v3–v11. 10.1093/rheumatology/kes113 22718924

[B5] Clark P. M. Dawany N. Dampier W. Byers S. W. Pestell R. G. Tozeren A. (2012). Bioinformatics analysis reveals transcriptome and microRNA signatures and drug repositioning targets for IBD and other autoimmune diseases. Inflamm. Bowel Dis. 18, 2315–2333. 10.1002/ibd.22958 22488912

[B6] Clough E. Barrett T. Wilhite S. E. Ledoux P. Evangelista C. Kim I. F. (2024). NCBI GEO: archive for gene expression and epigenomics data sets: 23-year update. Nucleic Acids Res. 52, D138–D144. 10.1093/nar/gkad965 37933855 PMC10767856

[B7] Combe B. (2007). Early rheumatoid arthritis: strategies for prevention and management. Best. Pract. Res. Clin. Rheumatol. 21, 27–42. 10.1016/j.berh.2006.08.011 17350542

[B8] Conforti A. Di Cola I. Pavlych V. Ruscitti P. Berardicurti O. Ursini F. (2021). Beyond the joints, the extra-articular manifestations in rheumatoid arthritis. Autoimmun Rev. 20 (2), 102735. 10.1016/j.autrev.2020.102735 33346115

[B9] Deshmukh R. (2023). Rheumatoid arthritis: pathophysiology, current therapeutic strategies and recent advances in targeted drug delivery system. Mater Today Commun. 35, 105877. 10.1016/j.mtcomm.2023.105877

[B10] Diboun I. Wernisch L. Orengo C. A. Koltzenburg M. (2006). Microarray analysis after RNA amplification can detect pronounced differences in gene expression using limma. BMC Genomics 7, 252. 10.1186/1471-2164-7-252 17029630 PMC1618401

[B11] Edgar R. Domrachev M. Lash A. E. (2002). Gene expression omnibus: NCBI gene expression and hybridization array data repository. Nucleic Acids Res. 30, 207–210. 10.1093/nar/30.1.207 11752295 PMC99122

[B12] Firestein G. S. (2003). Evolving concepts of rheumatoid arthritis. Nature 423, 356–361. 10.1038/nature01661 12748655

[B13] Firestein G. S. McInnes I. B. (2017). Immunopathogenesis of rheumatoid arthritis. Immunity 46, 183–196. 10.1016/j.immuni.2017.02.006 28228278 PMC5385708

[B14] Friedman J. Hastie T. Tibshirani R. (2010). Regularization paths for generalized Linear models *via* coordinate descent. J. Stat. Softw. 33, 1–22. 10.18637/jss.v033.i01 20808728 PMC2929880

[B15] Wickham H. (2016). Ggplot2. Cham: Springer International Publishing. 10.1007/978-3-319-24277-4

[B16] Han B. K. Kuzin I. Gaughan J. P. Olsen N. J. Bottaro A. (2016). Baseline CXCL10 and CXCL13 levels are predictive biomarkers for tumor necrosis factor inhibitor therapy in patients with moderate to severe rheumatoid arthritis: a pilot, prospective study. Arthritis Res. Ther. 18, 93. 10.1186/s13075-016-0995-0 27102921 PMC4840903

[B17] Inamo J. (2020). T peripheral helper cells signature genes in patients with rheumatoid arthritis. Clin. Rheumatol. 39, 2019–2021. 10.1007/s10067-020-05062-y 32236900

[B18] Kolde R. (2010). Pheatmap: Pretty Heatmaps. CRAN: Contributed Packages. 10.32614/CRAN.package.pheatmap

[B19] Kuhn M. (2008). Building predictive models in *R* using the caret package. J. Stat. Softw. 28(5), 1–26. 10.18637/jss.v028.i05 27774042

[B20] Li X. Zhang X. Liu T. Zhang G. Chen D. Lin S. (2025). Identification of immune characteristic biomarkers and therapeutic targets in cuproptosis for rheumatoid arthritis by integrated bioinformatics analysis and single-cell RNA sequencing analysis. Front. Med. (Lausanne) 12, 12. 10.3389/fmed.2025.1520400 40166070 PMC11955502

[B21] Lu Q. Jiang Y. Cang X. Pan J. Shen X. Tang R. (2025). Study of the immune infiltration and sonic hedgehog expression mechanism in synovial tissue of rheumatoid arthritis-related interstitial lung disease under machine learning CIBERSORT Algorithm. Mol. Biotechnol. 67, 3217–3233. 10.1007/s12033-024-01245-z 39110383

[B22] Newman A. M. Steen C. B. Liu C. L. Gentles A. J. Chaudhuri A. A. Scherer F. (2019). Determining cell type abundance and expression from bulk tissues with digital cytometry. Nat. Biotechnol. 37, 773–782. 10.1038/s41587-019-0114-2 31061481 PMC6610714

[B23] Paleolog E. M. (2002). Angiogenesis in rheumatoid arthritis. Arthritis Res. Ther. 4, S81–S90. 10.1186/ar575 12110126 PMC3240151

[B24] Pan Z. Zhu T. Liu Y. Zhang N. (2022). Role of the CXCL13/CXCR5 axis in autoimmune diseases. Front. Immunol. 13, 13. 10.3389/fimmu.2022.850998 35309354 PMC8931035

[B25] Reimand J. Isserlin R. Voisin V. Kucera M. Tannus-Lopes C. Rostamianfar A. (2019). Pathway enrichment analysis and visualization of omics data using g:Profiler, GSEA, cytoscape and EnrichmentMap. Nat. Protoc. 14, 482–517. 10.1038/s41596-018-0103-9 30664679 PMC6607905

[B26] Ritchie M. E. Phipson B. Wu D. Hu Y. Law C. W. Shi W. (2015). Limma powers differential expression analyses for RNA-sequencing and microarray studies. Nucleic Acids Res. 43, e47. 10.1093/nar/gkv007 25605792 PMC4402510

[B27] Robin X. Turck N. Hainard A. Tiberti N. Lisacek F. Sanchez J.-C. (2011). pROC: an open-source package for R and S+ to analyze and compare ROC curves. BMC Bioinforma. 12, 77. 10.1186/1471-2105-12-77 21414208 PMC3068975

[B28] Satam H. Joshi K. Mangrolia U. Waghoo S. Zaidi G. Rawool S. (2023). Next-Generation sequencing technology: current trends and advancements. Biol. (Basel) 12, 997. 10.3390/biology12070997 37508427 PMC10376292

[B29] Schellekens G. A. Visser H. De Jong B. A. W. Van Den Hoogen F. H. J. Hazes J. M. W. Breedveld F. C. (2000). The diagnostic properties of rheumatoid arthritis antibodies recognizing a cyclic citrullinated peptide. Arthritis Rheum. 43, 155–163. 10.1002/1529-0131(200001)43:1<155::AID-ANR20>3.0.CO;2-3 10643712

[B30] Shannon P. Markiel A. Ozier O. Baliga N. S. Wang J. T. Ramage D. (2003). Cytoscape: a software environment for integrated models of biomolecular interaction networks. Genome Res. 13, 2498–2504. 10.1101/gr.1239303 14597658 PMC403769

[B31] Smyth G. K. (2005). “Limma: linear models for microarray data,” in Bioinformatics and Computational Biology Solutions Using R and Bioconductor. Editors Gentleman R. Carey V. J. Huber W. Irizarry R. A. Dudoit S. (New York, NY: Springer New York), 397–420. 10.1007/0-387-29362-0_23

[B32] Subramanian A. Tamayo P. Mootha V. K. Mukherjee S. Ebert B. L. Gillette M. A. (2005). Gene set enrichment analysis: a knowledge-based approach for interpreting genome-wide expression profiles. Proc. Natl. Acad. Sci. U. S. A. 102, 15545–15550. 10.1073/pnas.0506580102 16199517 PMC1239896

[B33] Szklarczyk D. Kirsch R. Koutrouli M. Nastou K. Mehryary F. Hachilif R. (2023). The STRING database in 2023: protein–protein association networks and functional enrichment analyses for any sequenced genome of interest. Nucleic Acids Res. 51, D638–D646. 10.1093/nar/gkac1000 36370105 PMC9825434

[B34] Wu Y.-K. Liu C.-D. Liu C. Wu J. Xie Z.-G. (2024). Machine learning and weighted gene co-expression network analysis identify a three-gene signature to diagnose rheumatoid arthritis. Front. Immunol. 15, 15. 10.3389/fimmu.2024.1387311 38711508 PMC11070572

[B35] Yang M. Zheng H. Su Y. Xu K. Yuan Q. Aihaiti Y. (2022). Bioinformatics analysis identified the hub genes, mRNA–miRNA–lncRNA axis, and signaling pathways involved in rheumatoid arthritis pathogenesis. Int. J. Gen. Med. 15, 3879–3893. 10.2147/IJGM.S353487 35422654 PMC9005080

[B36] Yu G. (2024). Thirteen years of clusterProfiler. Innovation 5, 100722. 10.1016/j.xinn.2024.100722 39529960 PMC11551487

[B37] Yu R. Zhang J. Zhuo Y. Hong X. Ye J. Tang S. (2021). Identification of diagnostic signatures and immune cell infiltration characteristics in rheumatoid arthritis by integrating bioinformatic analysis and machine-learning strategies. Front. Immunol. 12, 12. 10.3389/fimmu.2021.724934 34691030 PMC8526926

[B38] Yue S. Niu D. Ma W. Guan Y. Liu Q. Wang X. (2024). The CXCL10/CXCR3 axis regulates Th1 cell differentiation and migration in experimental autoimmune prostatitis through the PI3K/AKT pathway. Andrology 12, 1408–1418. 10.1111/andr.13571 38095276

[B39] Zhao L. Li Z. Zeng X. Xia C. Xu L. Xu Q. (2021). Circulating CD4 ^+^ FoxP3 ^–^ CXCR5 ^–^ CXCR3 ^+^ PD‐1 ^hi^ cells are elevated in active rheumatoid arthritis and reflect the severity of the disease. Int. J. Rheum. Dis. 24, 1032–1039. 10.1111/1756-185X.14170 34227243

[B40] Zhou S. Lu H. Xiong M. (2021). Identifying immune cell infiltration and effective diagnostic biomarkers in rheumatoid arthritis by bioinformatics analysis. Front. Immunol. 12, 12. 10.3389/fimmu.2021.726747 34484236 PMC8411707

[B41] Zlotnik A. Yoshie O. (2000). Chemokines: a new classification system and their role in immunity. Immunity 12, 121–127. 10.1016/S1074-7613(00)80165-X 10714678

