# Supplementary material for: Machine learning-based identification of CXCL13 and CXCL10 as key biomarkers in rheumatoid arthritis
Source: Front Bioinform. 2026 Oct 12;6:1925964. doi: 10.3389/fbinf.2026.1925964 (PMC13696930; doi:10.3389/fbinf.2026.1925964)
Supplement: Supplementary file 1 [file Supplementaryfile1.docx]

Supplementary Material

# 1 Supplementary Figures and Tables

## 1.1 Supplementary Figures

**Supplementary Figure 1.** Workflow of probe annotation and preprocessing of the three training microarray datasets.

## 1.2 Supplementary Tables

| **S.No** | **Preprocessing Step** | **GSE77298** | **GSE55457** | **GSE12021** |
| --- | --- | --- | --- | --- |
| 1. | Initial probes | 54,675 | 22,283 | 22,283 |
| 2. | Probes without gene symbols removed | 8,893 | 1,058 | 1,058 |
| 3. | Remaining annotated probes | 45,782 | 21,225 | 21,225 |
| 4. | Duplicate probes summarized (avereps) | 22,902 | 7,988 | 7,988 |
| 5. | Unique genes | 22,880 | 13,237 | 13,237 |
| 6. | Common genes retained | 13,237 | 13,237 | 13,237 |

**Supplementary Table 1.** Summary of probe annotation and preprocessing across the three training microarray datasets.

| **ONTOLOGY** | **Description** | **GeneRatio** | **pvalue** | **p.adjust** | **Count** |
| --- | --- | --- | --- | --- | --- |
| BP | leukocyte migration | 38/375 | 9.86E-16 | 2.02E-12 | 38 |
| BP | lymphocyte differentiation | 40/375 | 1.04E-15 | 2.02E-12 | 40 |
| BP | cell killing | 32/375 | 1.51E-15 | 2.02E-12 | 32 |
| BP | antigen receptor-mediated signaling pathway | 28/375 | 3.03E-15 | 3.05E-12 | 28 |
| BP | leukocyte chemotaxis | 27/375 | 2.63E-13 | 1.60E-10 | 27 |
| BP | killing of cells of another organism | 21/375 | 2.78E-13 | 1.60E-10 | 21 |
| BP | disruption of cell in another organism | 21/375 | 2.78E-13 | 1.60E-10 | 21 |
| BP | disruption of anatomical structure in another organism | 21/375 | 5.03E-13 | 2.53E-10 | 21 |
| BP | leukocyte cell-cell adhesion | 36/375 | 6.41E-13 | 2.87E-10 | 36 |
| BP | immune response-activating cell surface receptor signaling pathway | 32/375 | 8.98E-13 | 3.61E-10 | 32 |
| BP | leukocyte mediated immunity | 37/375 | 3.18E-12 | 1.16E-09 | 37 |
| BP | immune response-regulating cell surface receptor signaling pathway | 32/375 | 9.18E-12 | 2.94E-09 | 32 |
| BP | mononuclear cell migration | 25/375 | 9.49E-12 | 2.94E-09 | 25 |
| BP | chemotaxis | 35/375 | 2.66E-11 | 7.64E-09 | 35 |
| BP | taxis | 35/375 | 2.99E-11 | 8.01E-09 | 35 |
| BP | B cell receptor signaling pathway | 15/375 | 3.18E-11 | 8.01E-09 | 15 |
| BP | regulation of leukocyte migration | 24/375 | 4.93E-11 | 1.17E-08 | 24 |
| BP | T cell differentiation | 28/375 | 1.19E-10 | 2.36E-08 | 28 |
| BP | cell chemotaxis | 28/375 | 1.19E-10 | 2.36E-08 | 28 |
| BP | positive regulation of cell activation | 31/375 | 1.23E-10 | 2.36E-08 | 31 |
| BP | regulation of leukocyte cell-cell adhesion | 31/375 | 1.23E-10 | 2.36E-08 | 31 |
| BP | regulation of T cell activation | 31/375 | 1.48E-10 | 2.71E-08 | 31 |
| BP | positive regulation of leukocyte activation | 30/375 | 1.59E-10 | 2.78E-08 | 30 |
| BP | maintenance of location in cell | 22/375 | 2.78E-10 | 4.66E-08 | 22 |
| BP | maintenance of location | 22/375 | 7.33E-10 | 1.18E-07 | 22 |
| BP | positive regulation of leukocyte cell-cell adhesion | 25/375 | 8.11E-10 | 1.25E-07 | 25 |
| BP | response to chemokine | 15/375 | 9.28E-10 | 1.33E-07 | 15 |
| BP | cellular response to chemokine | 15/375 | 9.28E-10 | 1.33E-07 | 15 |
| BP | lymphocyte mediated immunity | 29/375 | 1.09E-09 | 1.52E-07 | 29 |
| BP | release of sequestered calcium ion into cytosol | 17/375 | 1.25E-09 | 1.68E-07 | 17 |
| BP | positive regulation of cell-cell adhesion | 27/375 | 1.36E-09 | 1.77E-07 | 27 |
| BP | negative regulation of sequestering of calcium ion | 17/375 | 1.41E-09 | 1.77E-07 | 17 |
| BP | regulation of sequestering of calcium ion | 17/375 | 1.77E-09 | 2.16E-07 | 17 |
| BP | myeloid leukocyte migration | 22/375 | 2.32E-09 | 2.75E-07 | 22 |
| BP | sequestering of calcium ion | 17/375 | 2.78E-09 | 3.20E-07 | 17 |
| BP | regulation of leukocyte chemotaxis | 16/375 | 4.24E-09 | 4.75E-07 | 16 |
| BP | alpha-beta T cell activation | 20/375 | 5.81E-09 | 6.32E-07 | 20 |
| BP | positive regulation of lymphocyte activation | 26/375 | 7.31E-09 | 7.74E-07 | 26 |
| BP | regulation of mononuclear cell migration | 17/375 | 8.05E-09 | 8.31E-07 | 17 |
| BP | humoral immune response | 24/375 | 1.31E-08 | 1.27E-06 | 24 |
| BP | leukocyte proliferation | 26/375 | 1.32E-08 | 1.27E-06 | 26 |
| BP | calcium ion transmembrane import into cytosol | 19/375 | 1.32E-08 | 1.27E-06 | 19 |
| BP | leukocyte mediated cytotoxicity | 17/375 | 1.61E-08 | 1.51E-06 | 17 |
| BP | positive regulation of T cell activation | 22/375 | 2.08E-08 | 1.90E-06 | 22 |
| BP | chemokine-mediated signaling pathway | 13/375 | 2.22E-08 | 1.98E-06 | 13 |
| BP | muscle system process | 30/375 | 4.36E-08 | 3.74E-06 | 30 |
| BP | granulocyte migration | 16/375 | 4.37E-08 | 3.74E-06 | 16 |
| BP | lymphocyte migration | 14/375 | 6.13E-08 | 5.14E-06 | 14 |
| BP | lymphocyte proliferation | 23/375 | 7.79E-08 | 6.40E-06 | 23 |
| BP | regulation of leukocyte differentiation | 24/375 | 1.08E-07 | 8.68E-06 | 24 |
| BP | regulation of release of sequestered calcium ion into cytosol | 12/375 | 1.14E-07 | 9.01E-06 | 12 |
| BP | mononuclear cell proliferation | 23/375 | 1.16E-07 | 9.01E-06 | 23 |
| BP | muscle contraction | 25/375 | 1.27E-07 | 9.62E-06 | 25 |
| BP | granulocyte chemotaxis | 14/375 | 1.31E-07 | 9.75E-06 | 14 |
| BP | regulation of lymphocyte differentiation | 19/375 | 1.52E-07 | 1.11E-05 | 19 |
| BP | negative regulation of leukocyte migration | 10/375 | 1.61E-07 | 1.16E-05 | 10 |
| BP | neutrophil migration | 13/375 | 1.85E-07 | 1.31E-05 | 13 |
| BP | calcium ion homeostasis | 23/375 | 2.26E-07 | 1.57E-05 | 23 |
| BP | regulation of chemotaxis | 18/375 | 3.00E-07 | 2.05E-05 | 18 |
| BP | positive regulation of release of sequestered calcium ion into cytosol | 8/375 | 3.34E-07 | 2.24E-05 | 8 |
| BP | regulation of T cell differentiation | 17/375 | 3.59E-07 | 2.37E-05 | 17 |
| BP | T cell migration | 11/375 | 4.47E-07 | 2.90E-05 | 11 |
| BP | dendritic cell chemotaxis | 7/375 | 5.52E-07 | 3.52E-05 | 7 |
| BP | regulation of inflammatory response | 27/375 | 5.93E-07 | 3.73E-05 | 27 |
| BP | neutrophil chemotaxis | 11/375 | 7.44E-07 | 4.61E-05 | 11 |
| BP | regulation of T cell migration | 9/375 | 7.63E-07 | 4.66E-05 | 9 |
| BP | intracellular calcium ion homeostasis | 21/375 | 8.39E-07 | 5.04E-05 | 21 |
| BP | regulation of immune effector process | 25/375 | 1.27E-06 | 7.50E-05 | 25 |
| BP | regulation of lymphocyte migration | 10/375 | 1.54E-06 | 8.95E-05 | 10 |
| BP | B cell activation | 20/375 | 1.66E-06 | 9.54E-05 | 20 |
| BP | regulation of antigen receptor-mediated signaling pathway | 10/375 | 1.99E-06 | 0.000113 | 10 |
| BP | positive regulation of leukocyte migration | 14/375 | 2.16E-06 | 0.00012 | 14 |
| BP | myofibril assembly | 10/375 | 2.25E-06 | 0.000124 | 10 |
| BP | T cell receptor signaling pathway | 14/375 | 2.33E-06 | 0.000127 | 14 |
| BP | calcium ion transport | 25/375 | 2.68E-06 | 0.000144 | 25 |
| BP | positive regulation of calcium ion transmembrane transport | 9/375 | 3.07E-06 | 0.000163 | 9 |
| BP | regulation of hemopoiesis | 25/375 | 3.15E-06 | 0.000164 | 25 |
| BP | dendritic cell migration | 7/375 | 3.18E-06 | 0.000164 | 7 |
| BP | regulation of calcium ion transmembrane transport | 14/375 | 3.41E-06 | 0.000174 | 14 |
| BP | membraneless organelle assembly | 25/375 | 3.55E-06 | 0.000178 | 25 |
| BP | regulation of leukocyte apoptotic process | 11/375 | 3.58E-06 | 0.000178 | 11 |
| BP | striated muscle contraction | 16/375 | 4.40E-06 | 0.000216 | 16 |
| BP | negative regulation of leukocyte chemotaxis | 6/375 | 4.50E-06 | 0.000218 | 6 |
| BP | calcium ion transmembrane transport | 21/375 | 4.73E-06 | 0.000227 | 21 |
| BP | positive regulation of lymphocyte differentiation | 13/375 | 4.92E-06 | 0.000233 | 13 |
| BP | adaptive immune response based on somatic recombination of immune receptors built from immunoglobulin superfamily domains | 23/375 | 6.27E-06 | 0.000293 | 23 |
| BP | positive regulation of transmembrane transport | 15/375 | 6.81E-06 | 0.000315 | 15 |
| BP | negative regulation of mononuclear cell migration | 7/375 | 7.15E-06 | 0.000323 | 7 |
| BP | positive regulation of T cell migration | 7/375 | 7.15E-06 | 0.000323 | 7 |
| BP | striated muscle cell differentiation | 20/375 | 7.32E-06 | 0.000327 | 20 |
| BP | positive regulation of T cell differentiation | 12/375 | 7.50E-06 | 0.000328 | 12 |
| BP | positive regulation of cation transmembrane transport | 12/375 | 7.50E-06 | 0.000328 | 12 |
| BP | regulation of T cell chemotaxis | 5/375 | 7.71E-06 | 0.00033 | 5 |
| BP | response to lipopolysaccharide | 21/375 | 7.74E-06 | 0.00033 | 21 |
| BP | B cell differentiation | 13/375 | 7.78E-06 | 0.00033 | 13 |
| BP | negative regulation of chemotaxis | 8/375 | 9.20E-06 | 0.000386 | 8 |
| BP | natural killer cell activation | 11/375 | 9.33E-06 | 0.000387 | 11 |
| BP | pyrimidine-containing compound transmembrane transport | 5/375 | 1.10E-05 | 0.00045 | 5 |
| BP | positive regulation of leukocyte differentiation | 15/375 | 1.12E-05 | 0.00045 | 15 |
| BP | positive regulation of hemopoiesis | 15/375 | 1.12E-05 | 0.00045 | 15 |
| BP | positive regulation of immune effector process | 18/375 | 1.13E-05 | 0.000451 | 18 |
| BP | positive regulation of monoatomic ion transmembrane transport | 12/375 | 1.21E-05 | 0.000479 | 12 |
| BP | natural killer cell mediated immunity | 10/375 | 1.32E-05 | 0.000514 | 10 |
| BP | regulation of leukocyte proliferation | 18/375 | 1.66E-05 | 0.000644 | 18 |
| BP | fat cell differentiation | 17/375 | 1.82E-05 | 0.000693 | 17 |
| BP | negative regulation of leukocyte apoptotic process | 8/375 | 1.82E-05 | 0.000693 | 8 |
| BP | T cell chemotaxis | 6/375 | 1.91E-05 | 0.00072 | 6 |
| BP | antimicrobial humoral response | 14/375 | 1.97E-05 | 0.000733 | 14 |
| BP | response to molecule of bacterial origin | 21/375 | 2.18E-05 | 0.000807 | 21 |
| BP | positive regulation of leukocyte chemotaxis | 10/375 | 2.32E-05 | 0.000848 | 10 |
| BP | positive regulation of lymphocyte migration | 7/375 | 2.36E-05 | 0.000856 | 7 |
| BP | positive regulation of mononuclear cell migration | 10/375 | 2.54E-05 | 0.000912 | 10 |
| BP | regulation of calcium ion transport | 16/375 | 2.65E-05 | 0.000944 | 16 |
| BP | regulation of neutrophil migration | 7/375 | 2.75E-05 | 0.000971 | 7 |
| BP | alpha-beta T cell differentiation | 12/375 | 2.94E-05 | 0.001028 | 12 |
| BP | negative regulation of cell adhesion | 19/375 | 3.12E-05 | 0.001084 | 19 |
| BP | regulation of monocyte chemotaxis | 6/375 | 3.47E-05 | 0.001195 | 6 |
| BP | positive regulation of calcium ion transport | 10/375 | 3.60E-05 | 0.001227 | 10 |
| BP | leukocyte apoptotic process | 11/375 | 4.06E-05 | 0.001374 | 11 |
| BP | nuclear division | 23/375 | 4.18E-05 | 0.001401 | 23 |
| BP | positive regulation of leukocyte proliferation | 13/375 | 4.42E-05 | 0.001471 | 13 |
| BP | NK T cell differentiation | 4/375 | 4.54E-05 | 0.001499 | 4 |
| BP | receptor clustering | 8/375 | 4.76E-05 | 0.001558 | 8 |
| BP | natural killer cell mediated cytotoxicity | 9/375 | 4.80E-05 | 0.001558 | 9 |
| BP | regulation of lymphocyte proliferation | 16/375 | 5.25E-05 | 0.001691 | 16 |
| BP | cell-substrate adhesion | 20/375 | 5.40E-05 | 0.001726 | 20 |
| BP | regulation of phosphatidylinositol 3-kinase/protein kinase B signal transduction | 18/375 | 5.51E-05 | 0.001745 | 18 |
| BP | cellular response to lipopolysaccharide | 15/375 | 5.76E-05 | 0.001812 | 15 |
| BP | striated muscle cell development | 13/375 | 5.94E-05 | 0.001852 | 13 |
| BP | response to nitric oxide | 5/375 | 6.03E-05 | 0.001867 | 5 |
| BP | positive regulation of inflammatory response | 13/375 | 6.29E-05 | 0.001929 | 13 |
| BP | regulation of mononuclear cell proliferation | 16/375 | 6.33E-05 | 0.001929 | 16 |
| BP | granzyme-mediated programmed cell death signaling pathway | 4/375 | 6.71E-05 | 0.002031 | 4 |
| BP | T cell differentiation in thymus | 9/375 | 6.91E-05 | 0.002069 | 9 |
| BP | regulation of monoatomic cation transmembrane transport | 17/375 | 6.94E-05 | 0.002069 | 17 |
| BP | sister chromatid segregation | 15/375 | 7.01E-05 | 0.002076 | 15 |
| BP | cellular defense response | 7/375 | 7.19E-05 | 0.002114 | 7 |
| BP | regulation of metal ion transport | 20/375 | 7.26E-05 | 0.002119 | 20 |
| BP | muscle cell differentiation | 22/375 | 7.93E-05 | 0.002295 | 22 |
| BP | regulation of T cell receptor signaling pathway | 7/375 | 8.15E-05 | 0.002326 | 7 |
| BP | regulation of granulocyte chemotaxis | 7/375 | 8.15E-05 | 0.002326 | 7 |
| BP | antimicrobial humoral immune response mediated by antimicrobial peptide | 11/375 | 8.22E-05 | 0.00233 | 11 |
| BP | regulation of leukocyte mediated immunity | 16/375 | 8.30E-05 | 0.002335 | 16 |
| BP | nuclear chromosome segregation | 18/375 | 8.94E-05 | 0.002498 | 18 |
| BP | monocyte chemotaxis | 7/375 | 9.20E-05 | 0.002555 | 7 |
| BP | B cell proliferation | 9/375 | 0.000106 | 0.002922 | 9 |
| BP | cellular extravasation | 8/375 | 0.000109 | 0.00298 | 8 |
| BP | positive regulation of cell development | 22/375 | 0.00011 | 0.002982 | 22 |
| BP | negative regulation of T cell apoptotic process | 5/375 | 0.000116 | 0.003108 | 5 |
| BP | regulation of lymphocyte chemotaxis | 5/375 | 0.000116 | 0.003108 | 5 |
| BP | myeloid leukocyte mediated immunity | 10/375 | 0.000124 | 0.003313 | 10 |
| BP | mitotic sister chromatid segregation | 13/375 | 0.000128 | 0.003384 | 13 |
| BP | cell-matrix adhesion | 15/375 | 0.000129 | 0.003384 | 15 |
| BP | striated muscle adaptation | 7/375 | 0.000131 | 0.003394 | 7 |
| BP | positive regulation of T cell chemotaxis | 4/375 | 0.000131 | 0.003394 | 4 |
| BP | regulation of chromosome separation | 8/375 | 0.000132 | 0.003394 | 8 |
| BP | cellular response to molecule of bacterial origin | 15/375 | 0.000135 | 0.003449 | 15 |
| BP | ossification involved in bone maturation | 5/375 | 0.000141 | 0.003594 | 5 |
| BP | positive regulation of monoatomic ion transport | 13/375 | 0.000142 | 0.003595 | 13 |
| BP | cellular response to ketone | 10/375 | 0.000143 | 0.003595 | 10 |
| BP | positive regulation of chemotaxis | 11/375 | 0.000156 | 0.003894 | 11 |
| BP | type II interferon production | 10/375 | 0.000164 | 0.004046 | 10 |
| BP | regulation of type II interferon production | 10/375 | 0.000164 | 0.004046 | 10 |
| BP | T cell proliferation | 14/375 | 0.000166 | 0.004073 | 14 |
| BP | mitotic nuclear division | 16/375 | 0.000171 | 0.004167 | 16 |
| BP | establishment of T cell polarity | 4/375 | 0.000176 | 0.004279 | 4 |
| BP | muscle organ development | 19/375 | 0.000185 | 0.004463 | 19 |
| BP | regulation of T cell apoptotic process | 6/375 | 0.000194 | 0.004657 | 6 |
| BP | positive regulation of JNK cascade | 9/375 | 0.000199 | 0.004731 | 9 |
| BP | regulation of T cell differentiation in thymus | 5/375 | 0.000204 | 0.004802 | 5 |
| BP | gamma-delta T cell activation | 5/375 | 0.000204 | 0.004802 | 5 |
| BP | immunoglobulin mediated immune response | 13/375 | 0.000222 | 0.005203 | 13 |
| BP | regulation of monoatomic ion transmembrane transport | 17/375 | 0.000228 | 0.005316 | 17 |
| BP | establishment of lymphocyte polarity | 4/375 | 0.000232 | 0.005358 | 4 |
| BP | bone maturation | 5/375 | 0.000243 | 0.005578 | 5 |
| BP | chromosome separation | 8/375 | 0.000244 | 0.005579 | 8 |
| BP | regulation of lymphocyte apoptotic process | 7/375 | 0.000247 | 0.005611 | 7 |
| BP | positive regulation of lymphocyte proliferation | 11/375 | 0.000249 | 0.005639 | 11 |
| BP | B cell mediated immunity | 13/375 | 0.000257 | 0.005737 | 13 |
| BP | muscle cell development | 13/375 | 0.000257 | 0.005737 | 13 |
| BP | regulation of monoatomic ion transport | 21/375 | 0.000277 | 0.006139 | 21 |
| BP | phosphatidylinositol 3-kinase/protein kinase B signal transduction | 18/375 | 0.000278 | 0.006139 | 18 |
| BP | positive regulation of mononuclear cell proliferation | 11/375 | 0.000279 | 0.006139 | 11 |
| BP | regulation of transmembrane transport | 21/375 | 0.000285 | 0.006229 | 21 |
| BP | positive regulation of antigen receptor-mediated signaling pathway | 5/375 | 0.000286 | 0.006229 | 5 |
| BP | heart contraction | 15/375 | 0.000289 | 0.006246 | 15 |
| BP | leukocyte activation involved in immune response | 17/375 | 0.000295 | 0.00634 | 17 |
| BP | muscle filament sliding | 4/375 | 0.000298 | 0.006384 | 4 |
| BP | regulation of bone remodeling | 6/375 | 0.00032 | 0.00682 | 6 |
| BP | cell activation involved in immune response | 17/375 | 0.000339 | 0.007188 | 17 |
| BP | lymphocyte chemotaxis | 6/375 | 0.00036 | 0.007585 | 6 |
| BP | bone remodeling | 8/375 | 0.000365 | 0.007648 | 8 |
| BP | leukocyte tethering or rolling | 5/375 | 0.000392 | 0.008167 | 5 |
| BP | regulation of cell killing | 9/375 | 0.000401 | 0.008311 | 9 |
| BP | cellular response to biotic stimulus | 15/375 | 0.000411 | 0.008486 | 15 |
| BP | heart process | 15/375 | 0.000427 | 0.008772 | 15 |
| BP | cellular component assembly involved in morphogenesis | 10/375 | 0.000444 | 0.008987 | 10 |
| BP | cellular anatomical entity morphogenesis | 10/375 | 0.000444 | 0.008987 | 10 |
| BP | CD4-positive, alpha-beta T cell activation | 10/375 | 0.000444 | 0.008987 | 10 |
| BP | sarcomere organization | 6/375 | 0.000451 | 0.009003 | 6 |
| BP | animal organ maturation | 5/375 | 0.000454 | 0.009003 | 5 |
| BP | regulation of neutrophil chemotaxis | 5/375 | 0.000454 | 0.009003 | 5 |
| BP | positive regulation of chromosome separation | 5/375 | 0.000454 | 0.009003 | 5 |
| BP | myoblast differentiation | 9/375 | 0.000456 | 0.009006 | 9 |
| BP | chromosome segregation | 20/375 | 0.000465 | 0.009062 | 20 |
| BP | actin-myosin filament sliding | 4/375 | 0.000471 | 0.009062 | 4 |
| BP | CD4-positive, alpha-beta T cell cytokine production | 4/375 | 0.000471 | 0.009062 | 4 |
| BP | regulation of respiratory burst | 4/375 | 0.000471 | 0.009062 | 4 |
| BP | positive regulation of lymphocyte chemotaxis | 4/375 | 0.000471 | 0.009062 | 4 |
| BP | regulation of T cell proliferation | 12/375 | 0.000479 | 0.009185 | 12 |
| BP | response to alcohol | 15/375 | 0.000516 | 0.009835 | 15 |
| BP | positive regulation of neutrophil migration | 5/375 | 0.000524 | 0.009944 | 5 |
| BP | cardiac myofibril assembly | 4/375 | 0.000579 | 0.010891 | 4 |
| BP | cellular response to prostaglandin E stimulus | 4/375 | 0.000579 | 0.010891 | 4 |
| BP | regulation of smooth muscle cell proliferation | 11/375 | 0.000675 | 0.012631 | 11 |
| BP | negative regulation of axonogenesis | 6/375 | 0.000685 | 0.012773 | 6 |
| BP | positive regulation of T cell receptor signaling pathway | 4/375 | 0.000704 | 0.013061 | 4 |
| BP | actin filament-based movement | 10/375 | 0.000729 | 0.013406 | 10 |
| BP | regulation of nuclear division | 10/375 | 0.000729 | 0.013406 | 10 |
| BP | positive regulation of smooth muscle cell proliferation | 8/375 | 0.000752 | 0.013766 | 8 |
| BP | meiotic nuclear division | 12/375 | 0.000785 | 0.014301 | 12 |
| BP | muscle tissue development | 20/375 | 0.000793 | 0.014311 | 20 |
| BP | response to ketone | 13/375 | 0.000793 | 0.014311 | 13 |
| BP | smooth muscle cell proliferation | 11/375 | 0.000816 | 0.014665 | 11 |
| BP | skeletal muscle contraction | 6/375 | 0.000834 | 0.014779 | 6 |
| BP | leukocyte adhesion to vascular endothelial cell | 6/375 | 0.000834 | 0.014779 | 6 |
| BP | positive regulation of protein serine/threonine kinase activity | 6/375 | 0.000834 | 0.014779 | 6 |
| BP | actin-mediated cell contraction | 9/375 | 0.00084 | 0.014825 | 9 |
| BP | membranous septum morphogenesis | 3/375 | 0.000843 | 0.014825 | 3 |
| BP | negative regulation of lymphocyte apoptotic process | 5/375 | 0.000885 | 0.015484 | 5 |
| BP | glucose metabolic process | 12/375 | 0.000892 | 0.015484 | 12 |
| BP | regulation of reproductive process | 12/375 | 0.000892 | 0.015484 | 12 |
| BP | negative regulation of protein depolymerization | 7/375 | 0.000914 | 0.015722 | 7 |
| BP | negative regulation of mitotic nuclear division | 6/375 | 0.000916 | 0.015722 | 6 |
| BP | regulation of leukocyte mediated cytotoxicity | 8/375 | 0.000918 | 0.015722 | 8 |
| BP | muscle adaptation | 9/375 | 0.000942 | 0.016068 | 9 |
| BP | regulation of cellular component size | 17/375 | 0.000962 | 0.01634 | 17 |
| BP | complement activation, classical pathway | 5/375 | 0.000999 | 0.016898 | 5 |
| BP | skeletal muscle adaptation | 4/375 | 0.00101 | 0.017003 | 4 |
| BP | striated muscle tissue development | 19/375 | 0.001054 | 0.017675 | 19 |
| BP | cardiac muscle contraction | 10/375 | 0.001096 | 0.018302 | 10 |
| BP | neural crest cell migration | 6/375 | 0.0011 | 0.018302 | 6 |
| BP | respiratory burst | 5/375 | 0.001124 | 0.018556 | 5 |
| BP | CD4-positive, CD25-positive, alpha-beta regulatory T cell differentiation | 3/375 | 0.001143 | 0.018556 | 3 |
| BP | positive regulation of T cell differentiation in thymus | 3/375 | 0.001143 | 0.018556 | 3 |
| BP | bronchus development | 3/375 | 0.001143 | 0.018556 | 3 |
| BP | cellular response to UV-A | 3/375 | 0.001143 | 0.018556 | 3 |
| BP | leukocyte degranulation | 7/375 | 0.001143 | 0.018556 | 7 |
| BP | negative regulation of organelle organization | 17/375 | 0.001151 | 0.018604 | 17 |
| BP | calcium-mediated signaling | 13/375 | 0.001166 | 0.01878 | 13 |
| BP | thymic T cell selection | 4/375 | 0.001193 | 0.019126 | 4 |
| BP | regulation of cell-matrix adhesion | 9/375 | 0.001241 | 0.019817 | 9 |
| BP | actomyosin structure organization | 12/375 | 0.00129 | 0.020526 | 12 |
| BP | negative regulation of neuron projection development | 9/375 | 0.001309 | 0.020544 | 9 |
| BP | heterotypic cell-cell adhesion | 6/375 | 0.001312 | 0.020544 | 6 |
| BP | T cell apoptotic process | 6/375 | 0.001312 | 0.020544 | 6 |
| BP | mesenchymal cell migration | 6/375 | 0.001312 | 0.020544 | 6 |
| BP | diencephalon development | 7/375 | 0.00132 | 0.02059 | 7 |
| BP | positive regulation of leukocyte mediated immunity | 10/375 | 0.001329 | 0.020651 | 10 |
| BP | developmental growth involved in morphogenesis | 13/375 | 0.001352 | 0.020934 | 13 |
| BP | positive regulation of phosphatidylinositol 3-kinase/protein kinase B signal transduction | 12/375 | 0.001396 | 0.021474 | 12 |
| BP | positive regulation of neutrophil chemotaxis | 4/375 | 0.001398 | 0.021474 | 4 |
| BP | positive regulation of T cell proliferation | 8/375 | 0.001418 | 0.021708 | 8 |
| BP | regulation of chromosome segregation | 9/375 | 0.001454 | 0.022163 | 9 |
| BP | positive regulation of transferase activity | 10/375 | 0.00146 | 0.02217 | 10 |
| BP | purine nucleobase transport | 3/375 | 0.001501 | 0.022462 | 3 |
| BP | lateral sprouting from an epithelium | 3/375 | 0.001501 | 0.022462 | 3 |
| BP | complement activation, GZMK pathway | 3/375 | 0.001501 | 0.022462 | 3 |
| BP | regulation of dendritic cell chemotaxis | 3/375 | 0.001501 | 0.022462 | 3 |
| BP | lymphocyte apoptotic process | 7/375 | 0.001517 | 0.022614 | 7 |
| BP | bone resorption | 6/375 | 0.001553 | 0.023063 | 6 |
| BP | female meiotic nuclear division | 5/375 | 0.001566 | 0.023082 | 5 |
| BP | regulatory T cell differentiation | 5/375 | 0.001566 | 0.023082 | 5 |
| BP | regulation of the force of heart contraction | 4/375 | 0.001626 | 0.02389 | 4 |
| BP | establishment of cell polarity | 10/375 | 0.001675 | 0.024367 | 10 |
| BP | T cell selection | 6/375 | 0.001685 | 0.024367 | 6 |
| BP | negative regulation of nuclear division | 6/375 | 0.001685 | 0.024367 | 6 |
| BP | mitotic spindle organization | 9/375 | 0.001695 | 0.024367 | 9 |
| BP | positive regulation of kinase activity | 9/375 | 0.001695 | 0.024367 | 9 |
| BP | spindle assembly | 9/375 | 0.001695 | 0.024367 | 9 |
| BP | release of sequestered calcium ion into cytosol by sarcoplasmic reticulum | 5/375 | 0.001738 | 0.024805 | 5 |
| BP | macrophage chemotaxis | 5/375 | 0.001738 | 0.024805 | 5 |
| BP | response to hypoxia | 15/375 | 0.001795 | 0.025536 | 15 |
| BP | complement activation | 6/375 | 0.001826 | 0.025881 | 6 |
| BP | negative regulation of protein-containing complex disassembly | 7/375 | 0.001855 | 0.025961 | 7 |
| BP | myeloid leukocyte activation | 13/375 | 0.001862 | 0.025961 | 13 |
| BP | cellular response to prostaglandin stimulus | 4/375 | 0.00188 | 0.025961 | 4 |
| BP | positive regulation of granulocyte chemotaxis | 4/375 | 0.00188 | 0.025961 | 4 |
| BP | positive regulation of protein targeting to membrane | 4/375 | 0.00188 | 0.025961 | 4 |
| BP | negative regulation of cytoskeleton organization | 10/375 | 0.001916 | 0.025961 | 10 |
| BP | nucleobase transport | 3/375 | 0.001923 | 0.025961 | 3 |
| BP | bone trabecula morphogenesis | 3/375 | 0.001923 | 0.025961 | 3 |
| BP | leukocyte aggregation | 3/375 | 0.001923 | 0.025961 | 3 |
| BP | dendritic cell apoptotic process | 3/375 | 0.001923 | 0.025961 | 3 |
| BP | regulation of dendritic cell apoptotic process | 3/375 | 0.001923 | 0.025961 | 3 |
| BP | collagen catabolic process | 5/375 | 0.001924 | 0.025961 | 5 |
| BP | dopamine metabolic process | 5/375 | 0.001924 | 0.025961 | 5 |
| BP | ameboidal-type cell migration | 13/375 | 0.001927 | 0.025961 | 13 |
| BP | forebrain development | 18/375 | 0.001929 | 0.025961 | 18 |
| BP | positive regulation of interleukin-1 beta production | 6/375 | 0.001976 | 0.026508 | 6 |
| BP | myeloid leukocyte differentiation | 13/375 | 0.001994 | 0.026665 | 13 |
| BP | meiotic cell cycle process | 12/375 | 0.002039 | 0.027171 | 12 |
| BP | negative regulation of supramolecular fiber organization | 10/375 | 0.002091 | 0.027782 | 10 |
| BP | negative chemotaxis | 5/375 | 0.002124 | 0.028026 | 5 |
| BP | release of sequestered calcium ion into cytosol by endoplasmic reticulum | 5/375 | 0.002124 | 0.028026 | 5 |
| BP | CD8-positive, alpha-beta T cell activation | 4/375 | 0.002159 | 0.028303 | 4 |
| BP | prostate gland epithelium morphogenesis | 4/375 | 0.002159 | 0.028303 | 4 |
| BP | negative regulation of locomotion | 18/375 | 0.002193 | 0.028656 | 18 |
| BP | negative regulation of cell migration | 17/375 | 0.002252 | 0.02933 | 17 |
| BP | regulation of cell-substrate adhesion | 12/375 | 0.002273 | 0.029511 | 12 |
| BP | musculoskeletal movement | 6/375 | 0.002303 | 0.0298 | 6 |
| BP | regulation of skeletal muscle adaptation | 3/375 | 0.002412 | 0.030814 | 3 |
| BP | bone mineralization involved in bone maturation | 3/375 | 0.002412 | 0.030814 | 3 |
| BP | spindle elongation | 3/375 | 0.002412 | 0.030814 | 3 |
| BP | neutrophil-mediated killing of bacterium | 3/375 | 0.002412 | 0.030814 | 3 |
| BP | positive regulation of T cell cytokine production | 4/375 | 0.002465 | 0.031205 | 4 |
| BP | response to prostaglandin E | 4/375 | 0.002465 | 0.031205 | 4 |
| BP | inhibitory synapse assembly | 4/375 | 0.002465 | 0.031205 | 4 |
| BP | regulation of morphogenesis of an epithelium | 6/375 | 0.00248 | 0.031297 | 6 |
| BP | negative regulation of cell growth | 10/375 | 0.002587 | 0.032542 | 10 |
| BP | positive regulation of production of molecular mediator of immune response | 9/375 | 0.002617 | 0.03274 | 9 |
| BP | regulation of mitotic nuclear division | 8/375 | 0.002619 | 0.03274 | 8 |
| BP | response to decreased oxygen levels | 15/375 | 0.002639 | 0.032889 | 15 |
| BP | regulation of lymphocyte mediated immunity | 11/375 | 0.002652 | 0.032946 | 11 |
| BP | positive regulation of leukocyte mediated cytotoxicity | 6/375 | 0.002668 | 0.032946 | 6 |
| BP | multicellular organismal movement | 6/375 | 0.002668 | 0.032946 | 6 |
| BP | antigen processing and presentation | 8/375 | 0.002759 | 0.033918 | 8 |
| BP | meiotic cell cycle | 14/375 | 0.002773 | 0.033918 | 14 |
| BP | regulation of canonical NF-kappaB signal transduction | 15/375 | 0.002794 | 0.033918 | 15 |
| BP | regulation of macrophage chemotaxis | 4/375 | 0.002801 | 0.033918 | 4 |
| BP | prostate gland morphogenesis | 4/375 | 0.002801 | 0.033918 | 4 |
| BP | establishment or maintenance of cell polarity | 12/375 | 0.002807 | 0.033918 | 12 |
| BP | mammary gland morphogenesis | 5/375 | 0.002815 | 0.033918 | 5 |
| BP | positive regulation of CD4-positive, alpha-beta T cell activation | 5/375 | 0.002815 | 0.033918 | 5 |
| BP | neural crest cell differentiation | 7/375 | 0.00287 | 0.034376 | 7 |
| BP | cellular response to interleukin-1 | 7/375 | 0.00287 | 0.034376 | 7 |
| BP | protein depolymerization | 8/375 | 0.002904 | 0.034688 | 8 |
| BP | phenol-containing compound catabolic process | 3/375 | 0.00297 | 0.034754 | 3 |
| BP | positive thymic T cell selection | 3/375 | 0.00297 | 0.034754 | 3 |
| BP | azole transmembrane transport | 3/375 | 0.00297 | 0.034754 | 3 |
| BP | morphogenesis of an epithelial bud | 3/375 | 0.00297 | 0.034754 | 3 |
| BP | response to UV-A | 3/375 | 0.00297 | 0.034754 | 3 |
| BP | regulation of thymocyte apoptotic process | 3/375 | 0.00297 | 0.034754 | 3 |
| BP | phospholipase C/protein kinase C signal transduction | 3/375 | 0.00297 | 0.034754 | 3 |
| BP | regulation of protein depolymerization | 7/375 | 0.003044 | 0.035489 | 7 |
| BP | extracellular matrix disassembly | 6/375 | 0.003076 | 0.035489 | 6 |
| BP | positive chemotaxis | 6/375 | 0.003076 | 0.035489 | 6 |
| BP | T cell costimulation | 5/375 | 0.003077 | 0.035489 | 5 |
| BP | T cell homeostasis | 5/375 | 0.003077 | 0.035489 | 5 |
| BP | regulation of JNK cascade | 9/375 | 0.003138 | 0.036084 | 9 |
| BP | spindle organization | 11/375 | 0.003194 | 0.036621 | 11 |
| BP | response to interleukin-1 | 8/375 | 0.003213 | 0.03674 | 8 |
| BP | monosaccharide metabolic process | 13/375 | 0.003255 | 0.03712 | 13 |
| BP | positive regulation of cell killing | 6/375 | 0.003297 | 0.037486 | 6 |
| BP | negative regulation of cell motility | 17/375 | 0.00331 | 0.037532 | 17 |
| BP | lymphocyte costimulation | 5/375 | 0.003357 | 0.037743 | 5 |
| BP | positive T cell selection | 5/375 | 0.003357 | 0.037743 | 5 |
| BP | sarcoplasmic reticulum calcium ion transport | 5/375 | 0.003357 | 0.037743 | 5 |
| BP | aminoglycan catabolic process | 4/375 | 0.003562 | 0.039616 | 4 |
| BP | negative regulation of microtubule depolymerization | 4/375 | 0.003562 | 0.039616 | 4 |
| BP | positive regulation of natural killer cell mediated cytotoxicity | 4/375 | 0.003562 | 0.039616 | 4 |
| BP | regulation of cell size | 10/375 | 0.003572 | 0.039616 | 10 |
| BP | hyaluronan catabolic process | 3/375 | 0.003602 | 0.039616 | 3 |
| BP | neutrophil-mediated killing of symbiont cell | 3/375 | 0.003602 | 0.039616 | 3 |
| BP | pyroptotic cell death | 3/375 | 0.003602 | 0.039616 | 3 |
| BP | negative regulation of lymphocyte migration | 3/375 | 0.003602 | 0.039616 | 3 |
| BP | humoral immune response mediated by circulating immunoglobulin | 5/375 | 0.003655 | 0.039865 | 5 |
| BP | microtubule depolymerization | 5/375 | 0.003655 | 0.039865 | 5 |
| BP | retinol metabolic process | 5/375 | 0.003655 | 0.039865 | 5 |
| BP | positive regulation of secretion by cell | 14/375 | 0.003828 | 0.041642 | 14 |
| BP | cellular component disassembly | 19/375 | 0.003876 | 0.042053 | 19 |
| BP | positive regulation of cell cycle process | 13/375 | 0.003914 | 0.042324 | 13 |
| BP | hormone metabolic process | 12/375 | 0.003922 | 0.042324 | 12 |
| BP | brown fat cell differentiation | 5/375 | 0.003971 | 0.042721 | 5 |
| BP | negative regulation of axon extension | 4/375 | 0.003991 | 0.042721 | 4 |
| BP | regulation of protein targeting to membrane | 4/375 | 0.003991 | 0.042721 | 4 |
| BP | positive regulation of alpha-beta T cell activation | 6/375 | 0.004029 | 0.042775 | 6 |
| BP | receptor-mediated endocytosis | 13/375 | 0.004034 | 0.042775 | 13 |
| BP | regulation of blood circulation | 13/375 | 0.004034 | 0.042775 | 13 |
| BP | regulation of extent of cell growth | 7/375 | 0.004038 | 0.042775 | 7 |
| BP | positive regulation of protein kinase activity | 8/375 | 0.004097 | 0.04328 | 8 |
| BP | temperature homeostasis | 10/375 | 0.004165 | 0.04389 | 10 |
| BP | positive regulation of cold-induced thermogenesis | 7/375 | 0.004264 | 0.044115 | 7 |
| BP | negative regulation of BMP signaling pathway | 6/375 | 0.004298 | 0.044115 | 6 |
| BP | positive regulation of type II interferon production | 6/375 | 0.004298 | 0.044115 | 6 |
| BP | positive regulation of interleukin-1 production | 6/375 | 0.004298 | 0.044115 | 6 |
| BP | regulation of myoblast differentiation | 6/375 | 0.004298 | 0.044115 | 6 |
| BP | xenobiotic transmembrane transport | 3/375 | 0.00431 | 0.044115 | 3 |
| BP | response to macrophage colony-stimulating factor | 3/375 | 0.00431 | 0.044115 | 3 |
| BP | negative regulation of bone remodeling | 3/375 | 0.00431 | 0.044115 | 3 |
| BP | actin filament severing | 3/375 | 0.00431 | 0.044115 | 3 |
| BP | cellular response to nitric oxide | 3/375 | 0.00431 | 0.044115 | 3 |
| BP | midbrain dopaminergic neuron differentiation | 3/375 | 0.00431 | 0.044115 | 3 |
| BP | hexose metabolic process | 12/375 | 0.004318 | 0.044115 | 12 |
| BP | regulation of T-helper 1 type immune response | 4/375 | 0.004453 | 0.045262 | 4 |
| BP | hyaluronan metabolic process | 4/375 | 0.004453 | 0.045262 | 4 |
| BP | JNK cascade | 10/375 | 0.00449 | 0.045522 | 10 |
| BP | T-helper 1 type immune response | 5/375 | 0.004661 | 0.04702 | 5 |
| BP | regulation of morphogenesis of a branching structure | 5/375 | 0.004661 | 0.04702 | 5 |
| BP | positive regulation of lipid localization | 7/375 | 0.004743 | 0.047638 | 7 |
| BP | muscle cell proliferation | 12/375 | 0.004746 | 0.047638 | 12 |
| BP | lipid catabolic process | 15/375 | 0.0048 | 0.048064 | 15 |
| BP | regulation of cell junction assembly | 13/375 | 0.004816 | 0.048097 | 13 |
| BP | positive regulation of phosphorylation | 12/375 | 0.005049 | 0.049841 | 12 |
| BP | positive regulation of T-helper 1 type immune response | 3/375 | 0.005097 | 0.049841 | 3 |
| BP | negative regulation of interleukin-12 production | 3/375 | 0.005097 | 0.049841 | 3 |
| BP | cell-cell adhesion mediated by integrin | 3/375 | 0.005097 | 0.049841 | 3 |
| BP | positive regulation of Rac protein signal transduction | 3/375 | 0.005097 | 0.049841 | 3 |
| BP | cellular response to leptin stimulus | 3/375 | 0.005097 | 0.049841 | 3 |
| BP | neutrophil mediated cytotoxicity | 3/375 | 0.005097 | 0.049841 | 3 |
| BP | cellular response to reactive nitrogen species | 3/375 | 0.005097 | 0.049841 | 3 |
| BP | anatomical structure maturation | 13/375 | 0.005102 | 0.049841 | 13 |
| CC | external side of plasma membrane | 33/387 | 1.74E-12 | 6.80E-10 | 33 |
| CC | sarcomere | 23/387 | 7.08E-11 | 1.38E-08 | 23 |
| CC | myofibril | 23/387 | 4.33E-10 | 5.63E-08 | 23 |
| CC | contractile muscle fiber | 23/387 | 9.59E-10 | 9.35E-08 | 23 |
| CC | I band | 14/387 | 1.39E-06 | 0.000109 | 14 |
| CC | alpha-beta T cell receptor complex | 5/387 | 1.90E-06 | 0.00012 | 5 |
| CC | Z disc | 13/387 | 2.15E-06 | 0.00012 | 13 |
| CC | IgG immunoglobulin complex | 5/387 | 3.04E-06 | 0.000148 | 5 |
| CC | IgM immunoglobulin complex | 4/387 | 2.67E-05 | 0.001158 | 4 |
| CC | mitotic spindle | 13/387 | 0.000112 | 0.004377 | 13 |
| CC | myofilament | 5/387 | 0.000126 | 0.004468 | 5 |
| CC | immunoglobulin complex | 12/387 | 0.00014 | 0.004556 | 12 |
| CC | immunological synapse | 6/387 | 0.000317 | 0.009497 | 6 |
| CC | striated muscle thin filament | 4/387 | 0.000774 | 0.020438 | 4 |
| CC | IgA immunoglobulin complex | 3/387 | 0.000786 | 0.020438 | 3 |
| CC | cell leading edge | 19/387 | 0.000991 | 0.024152 | 19 |
| CC | proteasome core complex, beta-subunit complex | 3/387 | 0.001065 | 0.024441 | 3 |
| CC | A band | 5/387 | 0.001131 | 0.024506 | 5 |
| CC | immunoglobulin complex, circulating | 3/387 | 0.0014 | 0.028739 | 3 |
| CC | clathrin-coated vesicle membrane | 9/387 | 0.001672 | 0.029362 | 9 |
| CC | membrane raft | 14/387 | 0.00174 | 0.029362 | 14 |
| CC | plasma membrane raft | 8/387 | 0.001821 | 0.029362 | 8 |
| CC | membrane microdomain | 14/387 | 0.001854 | 0.029362 | 14 |
| CC | plasma membrane signaling receptor complex | 15/387 | 0.001871 | 0.029362 | 15 |
| CC | GABA-ergic synapse | 8/387 | 0.001924 | 0.029362 | 8 |
| CC | endocytic vesicle | 16/387 | 0.001957 | 0.029362 | 16 |
| CC | blood microparticle | 9/387 | 0.002127 | 0.030371 | 9 |
| CC | cytolytic granule | 3/387 | 0.002251 | 0.030371 | 3 |
| CC | M band | 4/387 | 0.002258 | 0.030371 | 4 |
| CC | clathrin-coated endocytic vesicle | 7/387 | 0.002364 | 0.03073 | 7 |
| CC | muscle myosin complex | 3/387 | 0.002773 | 0.034889 | 3 |
| CC | sarcoplasm | 7/387 | 0.003167 | 0.037885 | 7 |
| CC | neuronal cell body | 19/387 | 0.003206 | 0.037885 | 19 |
| CC | clathrin-coated endocytic vesicle membrane | 6/387 | 0.003583 | 0.041095 | 6 |
| CC | extracellular matrix | 19/387 | 0.003992 | 0.044183 | 19 |
| CC | external encapsulating structure | 19/387 | 0.004078 | 0.044183 | 19 |
| CC | tertiary granule lumen | 5/387 | 0.00421 | 0.044372 | 5 |
| MF | glycosaminoglycan binding | 21/367 | 1.27E-08 | 4.86E-06 | 21 |
| MF | antigen binding | 18/367 | 1.51E-08 | 4.86E-06 | 18 |
| MF | chemokine activity | 10/367 | 3.91E-08 | 8.39E-06 | 10 |
| MF | cytokine activity | 19/367 | 2.67E-07 | 4.30E-05 | 19 |
| MF | immunoglobulin receptor binding | 6/367 | 1.21E-06 | 0.000156 | 6 |
| MF | chemokine receptor binding | 10/367 | 2.60E-06 | 0.00028 | 10 |
| MF | CXCR chemokine receptor binding | 5/367 | 1.46E-05 | 0.001341 | 5 |
| MF | endopeptidase activity | 22/367 | 1.95E-05 | 0.001567 | 22 |
| MF | G protein-coupled receptor binding | 18/367 | 2.98E-05 | 0.002115 | 18 |
| MF | heparin binding | 13/367 | 3.28E-05 | 0.002115 | 13 |
| MF | immune receptor activity | 12/367 | 6.60E-05 | 0.003866 | 12 |
| MF | actin binding | 22/367 | 7.68E-05 | 0.00412 | 22 |
| MF | cytokine receptor activity | 9/367 | 0.000124 | 0.005748 | 9 |
| MF | cytokine binding | 11/367 | 0.000125 | 0.005748 | 11 |
| MF | serine-type peptidase activity | 13/367 | 0.000141 | 0.006051 | 13 |
| MF | structural constituent of muscle | 6/367 | 0.000183 | 0.0072 | 6 |
| MF | serine hydrolase activity | 13/367 | 0.00019 | 0.0072 | 13 |
| MF | serine-type endopeptidase activity | 12/367 | 0.000217 | 0.007772 | 12 |
| MF | fibronectin binding | 5/367 | 0.000272 | 0.009222 | 5 |
| MF | metallopeptidase activity | 12/367 | 0.00031 | 0.009978 | 12 |
| MF | cytokine receptor binding | 15/367 | 0.000426 | 0.013067 | 15 |
| MF | CCR chemokine receptor binding | 6/367 | 0.000474 | 0.013874 | 6 |
| MF | purine nucleobase transmembrane transporter activity | 3/367 | 0.000817 | 0.021703 | 3 |
| MF | sulfur compound binding | 14/367 | 0.000837 | 0.021703 | 14 |
| MF | protein tyrosine kinase activator activity | 5/367 | 0.000843 | 0.021703 | 5 |
| MF | nucleobase transmembrane transporter activity | 3/367 | 0.001107 | 0.026334 | 3 |
| MF | hyaluronic acid binding | 4/367 | 0.001145 | 0.026334 | 4 |
| MF | semaphorin receptor binding | 4/367 | 0.001145 | 0.026334 | 4 |
| MF | SH2 domain binding | 5/367 | 0.001199 | 0.026621 | 5 |
| MF | kinase activator activity | 10/367 | 0.001283 | 0.027533 | 10 |
| MF | coreceptor activity | 6/367 | 0.00135 | 0.028048 | 6 |
| MF | metalloendopeptidase activity | 8/367 | 0.001403 | 0.02824 | 8 |
| MF | cargo receptor activity | 6/367 | 0.001727 | 0.033702 | 6 |
| MF | chemorepellent activity | 4/367 | 0.002074 | 0.03928 | 4 |
| MF | azole transmembrane transporter activity | 3/367 | 0.002337 | 0.043001 | 3 |

Supplementary table 2. GO enrichment analysis of differentially expressed genes. BP, CC, and MF terms are shown with Gene Ratio, gene count, *p* value, and BH-adjusted *p* value.

| ID | Description | pvalue | p.adjust | Count |
| --- | --- | --- | --- | --- |
| hsa04820 | Cytoskeleton in muscle cells | 3.85E-09 | 1.09E-06 | 24 |
| hsa04060 | Cytokine-cytokine receptor interaction | 2.77E-08 | 3.91E-06 | 26 |
| hsa04062 | Chemokine signaling pathway | 7.39E-08 | 6.95E-06 | 20 |
| hsa04061 | Viral protein interaction with cytokine and cytokine receptor | 1.79E-07 | 1.01E-05 | 14 |
| hsa04640 | Hematopoietic cell lineage | 1.79E-07 | 1.01E-05 | 14 |
| hsa05340 | Primary immunodeficiency | 2.99E-07 | 1.40E-05 | 9 |
| hsa04514 | Cell adhesion molecule (CAM) interaction | 0.000193 | 0.007756 | 13 |
| hsa04672 | Intestinal immune network for IgA production | 0.000232 | 0.008163 | 7 |
| hsa04659 | Th17 cell differentiation | 0.000404 | 0.012661 | 10 |
| hsa04662 | B cell receptor signaling pathway | 0.000451 | 0.012729 | 9 |
| hsa05166 | Human T-cell leukemia virus 1 infection | 0.000522 | 0.013385 | 15 |
| hsa04670 | Leukocyte transendothelial migration | 0.000663 | 0.014564 | 10 |
| hsa03320 | PPAR signaling pathway | 0.000671 | 0.014564 | 8 |
| hsa05410 | Hypertrophic cardiomyopathy | 0.000838 | 0.016878 | 9 |
| hsa04517 | IgSF CAM signaling | 0.001483 | 0.027882 | 17 |
| hsa05416 | Viral myocarditis | 0.001816 | 0.032012 | 7 |
| hsa04658 | Th1 and Th2 cell differentiation | 0.002305 | 0.038228 | 8 |
| hsa04910 | Insulin signaling pathway | 0.002488 | 0.038974 | 10 |
| hsa05323 | Rheumatoid arthritis | 0.002635 | 0.039111 | 8 |
| hsa05418 | Fluid shear stress and atherosclerosis | 0.003064 | 0.0432 | 10 |
| hsa05140 | Leishmaniasis | 0.003379 | 0.045379 | 7 |
| hsa04152 | AMPK signaling pathway | 0.003589 | 0.046003 | 9 |

Supplementary table 3. KEGG pathway enrichment analysis of differentially expressed genes. Enriched pathways are shown with Gene Ratio, gene count, *p* value, and BH-adjusted *p* value.

| ID | Description | setSize | enrichmentScore | NES | pvalue | p.adjust | qvalue | rank |
| --- | --- | --- | --- | --- | --- | --- | --- | --- |
| hsa05340 | Primary immunodeficiency | 34 | 0.824642 | 2.729481 | 1.00E-10 | 1.09E-08 | 7.39E-09 | 647 |
| hsa04061 | Viral protein interaction with cytokine and cytokine receptor | 89 | 0.650756 | 2.560299 | 1.00E-10 | 1.09E-08 | 7.39E-09 | 1246 |
| hsa05323 | Rheumatoid arthritis | 83 | 0.65259 | 2.528014 | 1.23E-10 | 1.09E-08 | 7.39E-09 | 1244 |
| hsa04060 | Cytokine-cytokine receptor interaction | 252 | 0.489366 | 2.221081 | 1.00E-10 | 1.09E-08 | 7.39E-09 | 1246 |
| hsa04062 | Chemokine signaling pathway | 171 | 0.523076 | 2.283838 | 1.61E-10 | 1.14E-08 | 7.73E-09 | 1250 |
| hsa04640 | Hematopoietic cell lineage | 94 | 0.620649 | 2.450654 | 4.68E-10 | 2.44E-08 | 1.66E-08 | 1148 |
| hsa04820 | Cytoskeleton in muscle cells | 204 | -0.47773 | -2.07561 | 4.84E-10 | 2.44E-08 | 1.66E-08 | 1329 |
| hsa04260 | Cardiac muscle contraction | 72 | -0.61104 | -2.26874 | 8.01E-09 | 3.54E-07 | 2.40E-07 | 1675 |
| hsa05169 | Epstein-Barr virus infection | 191 | 0.478302 | 2.121212 | 1.27E-08 | 4.97E-07 | 3.38E-07 | 1646 |
| hsa05414 | Dilated cardiomyopathy | 94 | -0.54675 | -2.14036 | 1.24E-07 | 4.37E-06 | 2.97E-06 | 2696 |
| hsa04672 | Intestinal immune network for IgA production | 42 | 0.707844 | 2.407778 | 1.86E-07 | 5.97E-06 | 4.06E-06 | 1116 |
| hsa05330 | Allograft rejection | 35 | 0.735227 | 2.462873 | 3.36E-07 | 9.12E-06 | 6.20E-06 | 1495 |
| hsa05410 | Hypertrophic cardiomyopathy | 90 | -0.5447 | -2.11435 | 3.22E-07 | 9.12E-06 | 6.20E-06 | 2696 |
| hsa04659 | Th17 cell differentiation | 100 | 0.53958 | 2.156445 | 4.02E-07 | 1.01E-05 | 6.89E-06 | 1267 |
| hsa04658 | Th1 and Th2 cell differentiation | 85 | 0.570213 | 2.225007 | 5.08E-07 | 1.12E-05 | 7.62E-06 | 1298 |
| hsa05168 | Herpes simplex virus 1 infection | 163 | 0.460837 | 2.004506 | 4.96E-07 | 1.12E-05 | 7.62E-06 | 2000 |
| hsa04650 | Natural killer cell mediated cytotoxicity | 116 | 0.515038 | 2.123214 | 6.21E-07 | 1.22E-05 | 8.31E-06 | 1663 |
| hsa04514 | Cell adhesion molecule (CAM) interaction | 126 | 0.495337 | 2.083126 | 6.43E-07 | 1.22E-05 | 8.31E-06 | 1532 |
| hsa05152 | Tuberculosis | 165 | 0.460473 | 1.998084 | 6.58E-07 | 1.22E-05 | 8.31E-06 | 2010 |
| hsa04064 | NF-kappa B signaling pathway | 93 | 0.536765 | 2.115961 | 2.13E-06 | 3.76E-05 | 2.56E-05 | 1176 |
| hsa05320 | Autoimmune thyroid disease | 48 | 0.648763 | 2.262345 | 2.25E-06 | 3.79E-05 | 2.58E-05 | 1495 |
| hsa04923 | Regulation of lipolysis in adipocytes | 54 | -0.60159 | -2.11257 | 4.13E-06 | 6.63E-05 | 4.51E-05 | 2359 |
| hsa05332 | Graft-versus-host disease | 40 | 0.661106 | 2.238437 | 4.99E-06 | 7.65E-05 | 5.20E-05 | 1495 |
| hsa04940 | Type I diabetes mellitus | 41 | 0.67275 | 2.275513 | 5.92E-06 | 8.53E-05 | 5.80E-05 | 1495 |
| hsa05140 | Leishmaniasis | 73 | 0.558106 | 2.111113 | 6.04E-06 | 8.53E-05 | 5.80E-05 | 1126 |
| hsa05321 | Inflammatory bowel disease | 61 | 0.582943 | 2.109049 | 8.17E-06 | 0.000111 | 7.54E-05 | 1298 |
| hsa04145 | Phagocytosis | 223 | 0.395774 | 1.779631 | 9.21E-06 | 0.00012 | 8.19E-05 | 2772 |
| hsa04612 | Antigen processing and presentation | 73 | 0.544217 | 2.058577 | 1.51E-05 | 0.000178 | 0.000121 | 1681 |
| hsa04142 | Lysosome biogenesis | 196 | 0.404611 | 1.808586 | 1.50E-05 | 0.000178 | 0.000121 | 3170 |
| hsa04024 | cAMP signaling pathway | 197 | -0.40324 | -1.74781 | 1.49E-05 | 0.000178 | 0.000121 | 2540 |
| hsa04910 | Insulin signaling pathway | 125 | -0.45018 | -1.83146 | 1.83E-05 | 0.000208 | 0.000141 | 3131 |
| hsa04620 | Toll-like receptor signaling pathway | 98 | 0.4855 | 1.935141 | 3.08E-05 | 0.000331 | 0.000225 | 1799 |
| hsa04261 | Adrenergic signaling in cardiomyocytes | 134 | -0.44866 | -1.8445 | 3.09E-05 | 0.000331 | 0.000225 | 2694 |
| hsa04152 | AMPK signaling pathway | 107 | -0.48022 | -1.92999 | 3.39E-05 | 0.000352 | 0.00024 | 3365 |
| hsa04080 | Neuroactive ligand-receptor interaction | 313 | -0.35474 | -1.61617 | 4.22E-05 | 0.000426 | 0.000289 | 2865 |
| hsa04662 | B cell receptor signaling pathway | 78 | 0.51824 | 1.950038 | 4.40E-05 | 0.000431 | 0.000293 | 1033 |
| hsa04020 | Calcium signaling pathway | 225 | -0.38244 | -1.68214 | 4.58E-05 | 0.000437 | 0.000297 | 2377 |
| hsa04081 | Hormone signaling | 203 | -0.3812 | -1.65684 | 0.00011 | 0.001018 | 0.000692 | 3095 |
| hsa05170 | Human immunodeficiency virus 1 infection | 186 | 0.389048 | 1.730258 | 0.000128 | 0.001163 | 0.000791 | 2369 |
| hsa04920 | Adipocytokine signaling pathway | 66 | -0.51277 | -1.86376 | 0.000138 | 0.001221 | 0.00083 | 1892 |
| hsa05164 | Influenza A | 160 | 0.403415 | 1.754066 | 0.000201 | 0.001728 | 0.001175 | 1646 |
| hsa05412 | Arrhythmogenic right ventricular cardiomyopathy | 78 | -0.49008 | -1.86789 | 0.000253 | 0.002125 | 0.001444 | 2404 |
| hsa03010 | Ribosome | 137 | -0.41287 | -1.71157 | 0.000309 | 0.002539 | 0.001726 | 4183 |
| hsa04660 | T cell receptor signaling pathway | 113 | 0.427189 | 1.757865 | 0.000349 | 0.002803 | 0.001906 | 1089 |
| hsa05162 | Measles | 127 | 0.407544 | 1.718059 | 0.00047 | 0.00361 | 0.002454 | 1646 |
| hsa04382 | Cornified envelope formation | 146 | -0.39317 | -1.63637 | 0.00047 | 0.00361 | 0.002454 | 3740 |
| hsa04621 | NOD-like receptor signaling pathway | 150 | 0.373928 | 1.604081 | 0.000704 | 0.005288 | 0.003595 | 1799 |
| hsa04740 | Olfactory transduction | 70 | -0.46725 | -1.71992 | 0.000981 | 0.007212 | 0.004903 | 4212 |
| hsa00350 | Tyrosine metabolism | 32 | -0.5924 | -1.82867 | 0.001054 | 0.007438 | 0.005057 | 1615 |
| hsa05166 | Human T-cell leukemia virus 1 infection | 206 | 0.347467 | 1.555008 | 0.001036 | 0.007438 | 0.005057 | 1626 |
| hsa04964 | Proximal tubule bicarbonate reclamation | 22 | -0.64773 | -1.85377 | 0.0011 | 0.007617 | 0.005179 | 1326 |
| hsa05310 | Asthma | 28 | 0.614826 | 1.917685 | 0.001557 | 0.010368 | 0.007049 | 1277 |
| hsa03320 | PPAR signaling pathway | 67 | -0.46335 | -1.69506 | 0.001538 | 0.010368 | 0.007049 | 790 |
| hsa04022 | cGMP-PKG signaling pathway | 147 | -0.37996 | -1.58883 | 0.001789 | 0.011692 | 0.007949 | 3474 |
| hsa05235 | PD-L1 expression and PD-1 checkpoint pathway in cancer | 80 | 0.446494 | 1.700561 | 0.002102 | 0.013491 | 0.009172 | 853 |
| hsa04110 | Cell cycle | 138 | 0.378146 | 1.604856 | 0.002262 | 0.014259 | 0.009695 | 1455 |
| hsa04925 | Aldosterone synthesis and secretion | 85 | -0.42334 | -1.62113 | 0.00247 | 0.015035 | 0.010222 | 2540 |
| hsa04919 | Thyroid hormone signaling pathway | 113 | -0.39237 | -1.5764 | 0.002466 | 0.015035 | 0.010222 | 3388 |
| hsa04657 | IL-17 signaling pathway | 86 | 0.425286 | 1.652212 | 0.002662 | 0.015925 | 0.010827 | 1471 |
| hsa03030 | DNA replication | 34 | 0.56733 | 1.877804 | 0.003042 | 0.017025 | 0.011575 | 2732 |
| hsa04931 | Insulin resistance | 95 | -0.42242 | -1.65434 | 0.003087 | 0.017025 | 0.011575 | 1556 |
| hsa04613 | Neutrophil extracellular trap formation | 104 | 0.401762 | 1.614904 | 0.002911 | 0.017025 | 0.011575 | 1619 |
| hsa05417 | Lipid and atherosclerosis | 196 | 0.336316 | 1.503313 | 0.003071 | 0.017025 | 0.011575 | 1810 |
| hsa04714 | Thermogenesis | 168 | -0.35028 | -1.48021 | 0.003017 | 0.017025 | 0.011575 | 3918 |
| hsa05145 | Toxoplasmosis | 105 | 0.403022 | 1.627902 | 0.003287 | 0.017851 | 0.012137 | 1312 |
| hsa05150 | Staphylococcus aureus infection | 78 | 0.434 | 1.633058 | 0.003339 | 0.01786 | 0.012143 | 1365 |
| hsa04115 | p53 signaling pathway | 65 | 0.456447 | 1.686687 | 0.003922 | 0.020664 | 0.014049 | 1494 |
| hsa04670 | Leukocyte transendothelial migration | 100 | 0.392121 | 1.567121 | 0.004736 | 0.024587 | 0.016716 | 724 |
| hsa04922 | Glucagon signaling pathway | 89 | -0.41155 | -1.58897 | 0.005758 | 0.029455 | 0.020026 | 1064 |
| hsa04814 | Motor proteins | 150 | -0.34469 | -1.45136 | 0.006153 | 0.03103 | 0.021097 | 1368 |
| hsa04666 | Fc gamma R-mediated phagosome formation | 87 | 0.392715 | 1.53419 | 0.006371 | 0.031676 | 0.021536 | 2189 |
| hsa04211 | Longevity regulating pathway | 78 | -0.43161 | -1.64507 | 0.006621 | 0.032459 | 0.022069 | 1546 |
| hsa05033 | Nicotine addiction | 34 | -0.52775 | -1.66517 | 0.006796 | 0.032865 | 0.022344 | 2649 |
| hsa04068 | FoxO signaling pathway | 121 | -0.37392 | -1.51407 | 0.007492 | 0.035741 | 0.0243 | 1649 |
| hsa00620 | Pyruvate metabolism | 42 | -0.49845 | -1.66136 | 0.00772 | 0.036335 | 0.024703 | 271 |
| hsa05322 | Systemic lupus erythematosus | 49 | 0.480985 | 1.685506 | 0.007956 | 0.036954 | 0.025124 | 1126 |
| hsa04010 | MAPK signaling pathway | 279 | -0.30391 | -1.36796 | 0.008426 | 0.038626 | 0.026262 | 1698 |
| hsa04668 | TNF signaling pathway | 109 | 0.368025 | 1.497058 | 0.009259 | 0.041902 | 0.028489 | 1501 |
| hsa04213 | Longevity regulating pathway - multiple species | 54 | -0.45528 | -1.59877 | 0.009428 | 0.042127 | 0.028641 | 1546 |
| hsa04927 | Cortisol synthesis and secretion | 57 | -0.4504 | -1.60036 | 0.009717 | 0.042876 | 0.029151 | 2622 |
| hsa04350 | TGF-beta signaling pathway | 98 | -0.37527 | -1.48233 | 0.010485 | 0.045693 | 0.031066 | 2985 |
| hsa05030 | Cocaine addiction | 42 | -0.48696 | -1.62307 | 0.011234 | 0.048123 | 0.032718 | 2131 |
| hsa05135 | Yersinia infection | 127 | 0.345493 | 1.456475 | 0.011315 | 0.048123 | 0.032718 | 2144 |
| hsa04520 | Adherens junction | 83 | -0.40278 | -1.53954 | 0.011591 | 0.048712 | 0.033118 | 2258 |

Supplementary table 4. GSEA results. Significantly enriched gene sets are shown with gene-set size, NES, nominal *p* value, and FDR-adjusted *p* value.

| **Selected Genes** | **Coefficient** |
| --- | --- |
| CXCL13 | 0.330305 |
| CXCL10 | 0.286534 |

Supplementary Table 5. Genes selected by LASSO regression.

**Selected Genes**

CXCL13

CXCL10

Supplementary Table 6. Genes selected by SVM-RFE.

| **Dataset** | **Biomarker** | **AUC** | **Accuracy** | **Sensitivity** | **Specificity** |
| --- | --- | --- | --- | --- | --- |
| Training | CXCL13 | 0.954 | 0.896 | 0.854 | 0.962 |
| Training | CXCL10 | 0.925 | 0.881 | 0.878 | 0.885 |
| Validation (GSE89408) | CXCL13 | 0.901 | 0.817 | 0.809 | 0.857 |
| Validation (GSE89408) | CXCL10 | 0.886 | 0.789 | 0.757 | 0.964 |

**Supplementary Table 7.** Performance metrics of the identified hub genes in the training and validation datasets. Accuracy, sensitivity, specificity, precision, and F1 score of CXCL13 and CXCL10 in the training dataset and the independent validation dataset GSE89408.

| **Gene** | **Cell** | **Correlation** | **P_value** | **P_adj** |
| --- | --- | --- | --- | --- |
| CXCL10 | Macrophages M1 | 0.8331010 | 0 | 0 |
| CXCL13 | Macrophages M1 | 0.7519164 | 1.56E-07 | 3.27E-06 |
| CXCL13 | T cells gamma delta | 0.7175958 | 4.98E-07 | 6.97E-06 |
| CXCL13 | T cells CD4 naive | 0.6804235 | 9.87E-07 | 1.04E-05 |
| CXCL13 | T cells CD4 memory activated | 0.6458823 | 5.10E-06 | 4.29E-05 |
| CXCL13 | T cells follicular helper | 0.5759582 | 0.000109 | 0.000747 |
| CXCL13 | Macrophages M2 | -0.5716028 | 0.000125 | 0.000747 |
| CXCL13 | T cells CD8 | 0.5596350 | 0.000142 | 0.000747 |
| CXCL13 | Plasma cells | 0.5163801 | 0.000548 | 0.002557 |
| CXCL10 | T cells CD4 memory activated | 0.4770006 | 0.001615 | 0.006782 |
| CXCL10 | Plasma cells | 0.4422252 | 0.003792 | 0.014479 |
| CXCL10 | T cells gamma delta | 0.4372822 | 0.004586 | 0.014816 |
| CXCL10 | Macrophages M0 | -0.4355686 | 0.004422 | 0.014816 |
| CXCL13 | Mast cells activated | -0.4151990 | 0.006946 | 0.020839 |
| CXCL10 | T cells CD8 | 0.3985350 | 0.009855 | 0.027595 |
| CXCL10 | NK cells resting | -0.3733267 | 0.016209 | 0.042549 |
| CXCL13 | Dendritic cells resting | -0.3650896 | 0.018921 | 0.046746 |

Supplementary Table 8. Top significant correlations between hub gene expression and immune cell infiltration in rheumatoid arthritis samples.

| **Cell** | **p_value** | **p_adj** |
| --- | --- | --- |
| Plasma cells | 2.18E-06 | 2.40E-05 |
| T cells follicular helper | 2.29E-07 | 5.04E-06 |
| T cells gamma delta | 0.000125 | 0.000689 |
| Monocytes | 0.006272 | 0.022996 |
| Macrophages M1 | 0.000159 | 0.0007 |
| Mast cells resting | 3.06E-05 | 0.000224 |

Supplementary Table 9. Differentially infiltrated immune cell populations between rheumatoid arthritis and healthy control samples.

| **Group** | **Cell** | **Mean** | **SD** |
| --- | --- | --- | --- |
| RA | B cells naive | 0.02169 | 0.024901 |
| RA | B cells memory | 0.018782 | 0.024441 |
| RA | Plasma cells | 0.056703 | 0.08035 |
| RA | T cells CD8 | 0.016828 | 0.028679 |
| RA | T cells CD4 naive | 0.009852 | 0.014943 |
| RA | T cells CD4 memory resting | 0.052968 | 0.041909 |
| RA | T cells CD4 memory activated | 0.008497 | 0.011899 |
| RA | T cells follicular helper | 0.033249 | 0.02149 |
| RA | T cells regulatory (Tregs) | 0.007437 | 0.012388 |
| RA | T cells gamma delta | 0.089413 | 0.056805 |
| RA | NK cells resting | 0.001762 | 0.010877 |
| RA | NK cells activated | 0.01556 | 0.016661 |
| RA | Monocytes | 0.016362 | 0.02707 |
| RA | Macrophages M0 | 0.091459 | 0.113959 |
| RA | Macrophages M1 | 0.095876 | 0.052431 |
| RA | Macrophages M2 | 0.35402 | 0.130724 |
| RA | Dendritic cells resting | 0.01235 | 0.012028 |
| RA | Dendritic cells activated | 0 | 0 |
| RA | Mast cells resting | 0.059625 | 0.041372 |
| RA | Mast cells activated | 0.01591 | 0.038093 |
| RA | Eosinophils | 0.000789 | 0.002846 |
| RA | Neutrophils | 0.020866 | 0.045078 |

Supplementary Table 10. Mean immune cell fractions in rheumatoid arthritis samples

| **Gene** | **Cell** | **Correlation** | **P_value** | **P_adj** |
| --- | --- | --- | --- | --- |
| CXCL13 | B cells naive | 0.265678 | 0.093175 | 0.163056 |
| CXCL13 | B cells memory | 0.301487 | 0.055417 | 0.105795 |
| CXCL13 | Plasma cells | 0.51638 | 0.000548 | 0.002557 |
| CXCL13 | T cells CD8 | 0.559635 | 0.000142 | 0.000747 |
| CXCL13 | T cells CD4 naive | 0.680424 | 9.87E-07 | 1.04E-05 |
| CXCL13 | T cells CD4 memory resting | -0.31014 | 0.048447 | 0.096894 |
| CXCL13 | T cells CD4 memory activated | 0.645882 | 5.10E-06 | 4.29E-05 |
| CXCL13 | T cells follicular helper | 0.575958 | 0.000109 | 0.000747 |
| CXCL13 | T cells regulatory (Tregs) | -0.0193 | 0.904669 | 0.951648 |
| CXCL13 | T cells gamma delta | 0.717596 | 4.98E-07 | 6.97E-06 |
| CXCL13 | NK cells resting | -0.16846 | 0.292395 | 0.396148 |
| CXCL13 | NK cells activated | -0.23386 | 0.141119 | 0.22728 |
| CXCL13 | Monocytes | -0.04588 | 0.775759 | 0.951648 |
| CXCL13 | Macrophages M0 | -0.33966 | 0.029805 | 0.069544 |
| CXCL13 | Macrophages M1 | 0.751916 | 1.56E-07 | 3.27E-06 |
| CXCL13 | Macrophages M2 | -0.5716 | 0.000125 | 0.000747 |
| CXCL13 | Dendritic cells resting | -0.36509 | 0.018921 | 0.046746 |
| CXCL13 | Dendritic cells activated | NA | NA | NA |
| CXCL13 | Mast cells resting | -0.02111 | 0.89576 | 0.951648 |
| CXCL13 | Mast cells activated | -0.4152 | 0.006946 | 0.020839 |
| CXCL13 | Eosinophils | -0.20773 | 0.192481 | 0.288722 |
| CXCL13 | Neutrophils | 0.191845 | 0.229511 | 0.332395 |
| CXCL10 | B cells naive | -0.01436 | 0.92899 | 0.951648 |
| CXCL10 | B cells memory | 0.180246 | 0.259443 | 0.36322 |
| CXCL10 | Plasma cells | 0.442225 | 0.003792 | 0.014479 |
| CXCL10 | T cells CD8 | 0.398535 | 0.009855 | 0.027595 |
| CXCL10 | T cells CD4 naive | 0.259096 | 0.101884 | 0.171166 |
| CXCL10 | T cells CD4 memory resting | 0.041614 | 0.796151 | 0.951648 |
| CXCL10 | T cells CD4 memory activated | 0.477001 | 0.001615 | 0.006782 |
| CXCL10 | T cells follicular helper | 0.312718 | 0.04698 | 0.096894 |
| CXCL10 | T cells regulatory (Tregs) | -0.23105 | 0.146109 | 0.22728 |
| CXCL10 | T cells gamma delta | 0.437282 | 0.004586 | 0.014816 |
| CXCL10 | NK cells resting | -0.37333 | 0.016209 | 0.042549 |
| CXCL10 | NK cells activated | 0.105776 | 0.510409 | 0.669912 |
| CXCL10 | Monocytes | 0.02731 | 0.865407 | 0.951648 |
| CXCL10 | Macrophages M0 | -0.43557 | 0.004422 | 0.014816 |
| CXCL10 | Macrophages M1 | 0.833101 | 0 | 0 |
| CXCL10 | Macrophages M2 | -0.29512 | 0.061401 | 0.112124 |
| CXCL10 | Dendritic cells resting | 0.033342 | 0.836053 | 0.951648 |
| CXCL10 | Dendritic cells activated | NA | NA | NA |
| CXCL10 | Mast cells resting | 0.017623 | 0.912918 | 0.951648 |
| CXCL10 | Mast cells activated | -0.32254 | 0.039712 | 0.087785 |
| CXCL10 | Eosinophils | -0.00575 | 0.97153 | 0.97153 |
| CXCL10 | Neutrophils | 0.054365 | 0.735672 | 0.93631 |

Supplementary Table 11. Complete Spearman correlation analysis between hub gene expression and immune cell infiltration in rheumatoid arthritis.
